# The complete chloroplast genomes of three Hamamelidaceae species: Comparative and phylogenetic analyses

**DOI:** 10.1002/ece3.8637

**Published:** 2022-02-16

**Authors:** NingJie Wang, ShuiFei Chen, Lei Xie, Lu Wang, YueYao Feng, Ting Lv, YanMing Fang, Hui Ding

**Affiliations:** ^1^ 74584 Co‐Innovation Center for Sustainable Forestry in Southern China College of Biology and the Environment Key Laboratory of State Forestry and Grassland Administration on Subtropical Forest Biodiversity Conservation Nanjing Forestry University Nanjing China; ^2^ Research Center for Nature Conservation and Biodiversity State Environmental Protection Scientific Observation and Research Station for Ecology and Environment of Wuyi Mountains State Environmental Protection Key Laboratory on Biosafety Nanjing Institute of Environmental Sciences, Ministry of Ecology and Environment Nanjing China

**Keywords:** chloroplast genomes, comparative analysis, Hamamelidaceae, phylogenetic relationship

## Abstract

Hamamelidaceae is an important group that represents the origin and early evolution of angiosperms. Its plants have many uses, such as timber, medical, spice, and ornamental uses. In this study, the complete chloroplast genomes of *Loropetalum chinense* (R. Br.) Oliver, *Corylopsis glandulifera* Hemsl., and *Corylopsis velutina* Hand.‐Mazz. were sequenced using the Illumina NovaSeq 6000 platform. The sizes of the three chloroplast genomes were 159,402 bp (*C*. *glandulifera*), 159,414 bp (*C*. *velutina*), and 159,444 bp (*L*. *chinense*), respectively. These chloroplast genomes contained typical quadripartite structures with a pair of inverted repeat (IR) regions (26,283, 26,283, and 26,257 bp), a large single‐copy (LSC) region (88,134, 88,146, and 88,160 bp), and a small single‐copy (SSC) region (18,702, 18,702, and 18,770 bp). The chloroplast genomes encoded 132–133 genes, including 85–87 protein‐coding genes, 37–38 tRNA genes, and 8 rRNA genes. The coding regions were composed of 26,797, 26,574, and 26,415 codons, respectively, most of which ended in A/U. A total of 37–43 long repeats and 175–178 simple sequence repeats (SSRs) were identified, and the SSRs contained a higher number of A + T than G + C bases. The genome comparison showed that the IR regions were more conserved than the LSC or SSC regions, while the noncoding regions contained higher variability than the gene coding regions. Phylogenetic analyses revealed that species in the same genus tended to cluster together. *Chunia* Hung T. Chang, *Mytilaria* Lecomte, and *Disanthus* Maxim. may have diverged early and *Corylopsis* Siebold & Zucc. was closely related to *Loropetalum* R. Br. This study provides valuable information for further species identification, evolution, and phylogenetic studies of Hamamelidaceae plants.

## Graphical abstract

In this study, the complete cp genomes of three Hamamelidaceae species were determined by Illumina high‐throughput sequencing technology, and the basic structures, conservation, and variability of these sequences were revealed. This study provides valuable information for further researches on the species identification, evolution, and phylogeny of Hamamelidaceae plants.

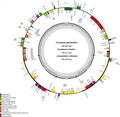

## 1 INTRODUCTION

Hamamelidaceae is an important group representing the origin and early evolution of angiosperms and is well known for its broad and scattered geographic distribution and endemics (Endress, 1993; Zhang & Lu, 1995). Hamamelidaceae fossils have been found in Upper Cretaceous‐early Tertiary strata (Manchester et al., 2009; Zhang & Lu, 1995); thus, the flora of Hamamelidaceae may have arisen earlier than the Cretaceous. This family contains 28 genera and about 120 species (Judd et al., 2007), which mainly occur in Eastern Asia, while others are distributed in the Americas, Africa, and Oceania. The plants in Hamamelidaceae are all woody, including *Liquidambar* L., *Altingia* Noronha, *Exbucklandia* R. W. Brown, *Chunia* Hung T. Chang, *Mytilaria* Lecomte, and *Semiliquidambar* Chang, which are used in the construction and furniture industries (Qin et al., 2019). *Liquidambar*, *Altingia*, *Semiliquidambar*, *Hamamelis* Gronov. ex L., *Fortunearia* Rehder & E. H. Wilson, and *Corylopsis* Siebold & Zucc. are used as medicinal materials (Kim et al., 2020; Simon et al., 2021). In addition, most of the genera have ornamental value, particularly *Rhodoleia* Champ. ex Hook. and *Corylopsis*.

The chloroplast is an important plant organelle and photosynthetic organ (Douglas, 1994). It is also a semiautonomous genetic organelle that contains independent chloroplast DNA (cpDNA), which has a length of 110–160 kb (Choi & Park, 2015). In general, cpDNA has a circular structure that includes one large single‐copy (LSC) region, one short single‐copy (SSC) region, and two inverted repeat (IR) regions, with the IR region separating the LSC and SSC regions (Ferrarini et al., 2013; Wu et al., 2014; Xue et al., 2019). The chloroplast genome is independent of the nuclear genome and corresponds to maternal inheritance with independent transcription and transport systems (Wu et al., 2020). Considering the similar structures, highly conserved sequences, and stable maternal heredity, the chloroplast genome has become an ideal resource for species identification, population genetics, phylogenetic, and genetic engineering studies (Fan et al., 2021; Nock et al., 2014). Moreover, gene mutations, rearrangements, duplications, and losses can be detected in the chloroplast genomes of the angiosperm lineages (Li et al., 2020; Luo et al., 2021). Structural changes in the genome can be used to study the taxonomic significance and phylogenetic relationships, and provide information for the development of genomic markers (Cheng et al., 2020; Watson et al., 2002). Repeat sequences are DNA sequence motifs that are repeated hundreds or thousands of times at different positions in the genome (Biscotti et al., 2015). They are ubiquitous in genomes and play important roles in evolution. Repeat sequences are mainly divided into two categories: one is tandem repeats, which mainly include some shorter repeats, such as simple sequence repeats (SSRs), and the other is interspersed repeated sequences, which are commonly known as transposons (Treangen & Salzberg, 2011). SSRs are composed of 1–6 nucleotide repeat units and are also called microsatellites, which have been widely used as molecular markers in population genetics and evolutionary biology (Bondar et al., 2019; Dashnow et al., 2015) due to their highly reproducible, codominance, multi‐allelic, and chromosome‐specific nature (Miri et al., 2014; Oliveira et al., 2006; Vieira et al., 2016). Interspersed repeated sequences account for most of the plant genomic repeats (Zhao & Ma, 2013), whereas retrotransposons play an important role in genome amplification (Ammiraju et al., 2007; Baucom et al., 2009; Paterson et al., 2009; SanMiguel et al., 2009; Schnable et al., 2009) and contribute to the expansion and contraction of the genome and the difference in the interspecific sequence (Morgante et al., 2007). The complete chloroplast genome contains all genes used to reconstruct the evolutionary history and provides more valuable and high‐quality information for evolutionary and phylogenetic analyses (Li et al., 2019). Complete chloroplast genome sequences are easily obtained due to the rapid development of large‐scale high‐throughput sequencing techniques, such as the Illumina and PacBio sequencing platforms (Huang et al., 2019; Kim et al., 2021; Lin et al., 2018; Yang et al., 2019; Ye et al., 2020).

Hamamelidaceae is a key family to study the phylogeny of angiosperms (Zhang et al., 2001). The relationships between genera in this family have been controversial for a long time (Hao & Wei, 1998; Li Bogle et al., 1999; Li Bogle et al., 1999; Li et al., 1997; Magallon, 2007; Xie et al., 2010). For example, Ye et al. (2020) reported that *Hamamelis* is sister to the clade that includes *Parrotia* C. A. Mey. and *Distylium* Siebold & Zucc., which is consistent with previous studies (Li, Bogle, & Donoghue, 1999; Li, Bogle, & Klein, 1999; Magallon, 2007; Shi et al., 1998; Xie et al., 2010). The results of another study showed that *Parrotia subaequalis* (H. T. Chang) R. M. Hao & H. T. Wei is in the *Distylium* genus (Chen et al., 2020), which is consistent with the result of Jiang et al. (2020). Different taxonomists have systematically divided Hamamelidaceae based on morphology, anatomy, and palynology (Bogle & Philbrick, 1980; Harms, 1930; Reinsch, 1890), but the traditional identification method based on morphological characteristics cannot be used to clearly distinguish Hamamelidaceae species (Deng et al., 1992; Endress, 1969, 1989; Zhang, 1999). In recent years, phylogenetic analyses of Hamamelidaceae species have been carried out with the rapid development of molecular technology (Li et al., 2000; Shi et al., 1998; Wen & Shi, 1999; Xiang et al., 2019; Xie et al., 2010; Zhou et al., 2019), and early studies focused on DNA fragment‐labeling techniques or phylogenetic analyses based on nuclear or chloroplast DNA fragments. However, limited nuclear or chloroplast DNA fragments do not provide sufficient phylogenetic information to effectively solve interspecific relationships (Hao & Wei, 1998; Li et al., 1997). Complete chloroplast genomes provide more valuable and higher‐quality information for evolutionary and phylogenetic analyses and reduce the sampling error inherent in studies of one or a few genes that may indicate critical evolutionary events (Cho et al., 2019). Thus, a clear phylogenetic relationship with Hamamelidaceae or the relationships between and within genera may be established based on conserved chloroplast genomes. Can the LSC, SSC, and IR regions of chloroplast genomes be used to establish a phylogenetic relationship within Hamamelidaceae?

In the present study, the complete chloroplast genomes of *Loropetalum chinense*, *Corylopsis glandulifera*, and *Corylopsis velutina* (Hamamelidaceae) were sequenced using Illumina technology, and their features were characterized. Our research purposes were to: (1) study the molecular structures of these three chloroplast genomes; (2) examine the variations in the repeat sequences and the SSRs in the three chloroplast genomes; (3) discover the divergence hotspot regions to provide potential molecular markers for future phylogenetic studies; and (4) establish and analyze the phylogenetic relationships of Hamamelidaceae species based on their complete chloroplast genome sequences, as well as the LSC, SSC, and IR regions. The data obtained in this study will provide valuable reference information for further studies on species identification and evolution, as well as population genetics and phylogenetic analyses of Hamamelidaceae.

## 2 MATERIALS AND METHODS

### 2.1 Plant material, DNA extraction, and sequencing

Fresh and healthy leaves of *L*. *chinense* and *C*. *velutina* were collected from the Nanjing Forestry University in Nanjing, Jiangsu, China (32°04′N, 118°48′E). Fresh and healthy leaves of *C*. *glandulifera* were collected from Mount Huang in Anhui, China (30°8′N, 118°6′E). All voucher specimens were deposited at the Herbarium of Nanjing Forestry University, Nanjing, Jiangsu, China with collection numbers 2021–20 (*L*. *chinense*), 2021–21 (*C*. *velutina*), and 2021–29 (*C*. *glandulifera*). After the quality inspection of the genomic DNA was performed, the DNA was fragmented by mechanical interruption (ultrasound). Then, the fragmented DNA was purified, end repaired, 3′ end plus A, connected to a sequencing adapter, and agarose gel electrophoresis was used to select the fragment size. The polymerase chain reaction (PCR) product was amplified to form the sequencing library. The qualified library was sequenced with the Illumina NovaSeq 6000 platform, and the sequencing read length was 150 bp. The whole genome was sequenced by Nanjing Genepioneer Biotechnologies Inc. (Nanjing, China).

### 2.2 Chloroplast genome assembly and annotation

Fastp v0.20.0 (https://github.com/OpenGene/fastp) was used to trim the raw reads, and the high‐quality clean data were obtained by removing the connector sequences and low‐quality reads (the filtering criteria are in the Appendix S1). Bowtie2 v2.2.4 (Langmead & Salzberg, 2012) was used to align the clean data with the chloroplast genome database built by Genepioneer Biotechnologies a in very sensitive local mode. SPAdes v3.10.1 (Bankevich et al., 2012) was used to acquire SEED sequences, and the contigs were obtained using the kmer iterative extend seed. The contig sequences were linked into scaffolds using SSPACE v2.0 (Acemel et al., 2016) and then used in Gapfiller v2.1.1 (Boetzer & Pirovano, 2012) to fill the gaps (Xiong et al., 2020) (the assembly process is in the Appendix S1). Two methods were used to annotate the chloroplast genomes to improve the accuracy of the annotation. First, protein‐coding genes were annotated using Prodigal v2.6.3 (https://www.github.com/hyattpd/Prodigal). rRNA was predicted using Hmmer v3.1b2 (Eddy, 2008), and tRNA was predicted using Aragorn v1.2.38 (Laslett & Canback, 2004). Second, the assembled sequences were compared using Blast v2.6 (McGinnis & Madden, 2004) according to the related species published at the NCBI (https://www.ncbi.nlm.nih.gov/). Then, the two annotation results were compared and manually corrected. The circular gene maps were visualized using OGDRAW v1.2 (Lohse et al., 2007). An analysis of GC content, codon usage, and relative synonymous codon usage (RSCU) values was conducted in MEGA7 (Kumar et al., 2016). The repetitive sequences and SSRs were determined using Vmatch v2.3.0 (http://www.vmatch.de/) (parameter settings: minimum length = 30 bp, hamming distance = 3) and MISA v1.0 (MIcroSAtellite identification tool, http://pgrc.ipk‐gatersleben.de/misa/misa.html) (parameters 1–8 [single base repeat 8 times or more], 2–5, 3–3, 4–3, 5–3, 6–3), respectively.

### 2.3 Genome comparison

Chloroplast genome sequences are often used to measure genetic diversity within a species, gene flow between species, and ancestral population size of separated sister species (Cavender et al., 2015). Therefore, it is necessary to understand the divergence of chloroplasts between species. The online comparison tool mVISTA (Mayor et al., 2000) was applied to compare the whole chloroplast genomes of *L*. *chinense*, *C*. *glandulifera*, and *C*. *velutina* to three published chloroplast genomes of *Chunia bucklandioides* Chang (NC_041163), *Distylium tsiangii* Chun ex Walker (MN711651), and *Rhodoleia championii* Hook. f. (NC_045276) in Shuffle‐LAGAN mode (Frazer et al., 2004) with the *L*. *chinense* annotation as the reference. Although the IR regions are the most conserved, expansion and contraction of the IR boundary are the main reasons for differences in the sizes of chloroplast genomes (Kode et al., 2005; Raubeson et al., 2007; Yao et al., 2015). Irscope (Ali et al., 2018) was used to compare and visualize the borders of the LSC, SSC, and IR regions among the six Hamamelidaceae species. The six chloroplast genome sequences were aligned using MAFFT (Katoh & Standley, 2013) under default parameters, and then DnaSP v5.10 (Librado & Rozas, 2009) was utilized to detect nucleotide diversity (*Pi*). *Pi* values were calculated with a step size of 200 bp and a sliding window of 600 bp.

### 2.4 Phylogenetic analysis

To investigate the phylogenetic positions of *L*. *chinense*, *C*. *glandulifera*, and *C*. *velutina* within the Hamamelidaceae lineages, 28 complete chloroplast genome sequences (25 Hamamelidaceae species) were downloaded from the NCBI GenBank, along with *Altingia chinensis* (Champ.) Oliver ex Hance, *Liquidambar formosana* Hance, and *Cercidiphyllum japonicum* Sieb. et Zucc. as outgroups (Table S1). Maximum likelihood (ML) and Bayesian inference (BI) methods were used to perform phylogenetic analyses based on the following four datasets: (1) the complete chloroplast genome sequences; (2) LSC regions of the chloroplast genomes; (3) SSC regions of the chloroplast genomes; and (4) IR regions of the chloroplast genomes. The ML analysis (Guindon et al., 2010) was conducted using IQ‐TREE (Nguyen et al., 2015) and Ultrafast bootstrap (Minh et al., 2013), and the BI analysis was conducted using MrBayes (Ronquist et al., 2012). All datasets were aligned using MAFFT (Katoh & Standley, 2013) under default parameters. ModelFinder (Kalyaanamoorthy et al., 2017) was used to select the best‐fit model using Akaike's Information Criterion and GTR (general time‐reversible)+F+I+G4 was selected as the best substitution model for the complete chloroplast genome sequences and the LSC regions. GTR+F+G4 was selected as the best substitution model for the SSC regions and GTR+F+I was selected for the IR regions. The ML analysis was conducted with 1,000 repetitions of Ultrafast bootstrap and 1,000 bootstrap replicates of the Shimodaira/Hasegawa approximate likelihood‐ratio test (SH‐aLRT) (Guindon et al., 2010). The Markov chain Monte Carlo algorithms were run for 2,000,000 generations and sampled every 100 generations for the BI analysis. The first 25% of the generations were discarded as burn‐in. MAFFT, ModelFinder, IQ‐TREE, Ultrafast bootstrap, and MrBayes were used in PhyloSuite (Zhang, Gao, et al., 2020; Zhang, Wang, et al., 2020). The phylogenetic relationships were visualized using FigTree (http://tree.bio.ed.ac.uk/software/figtree/).

## 3 RESULTS

### 3.1 Chloroplast genome features of the three Hamamelidaceae species

**FIGURE 1:**
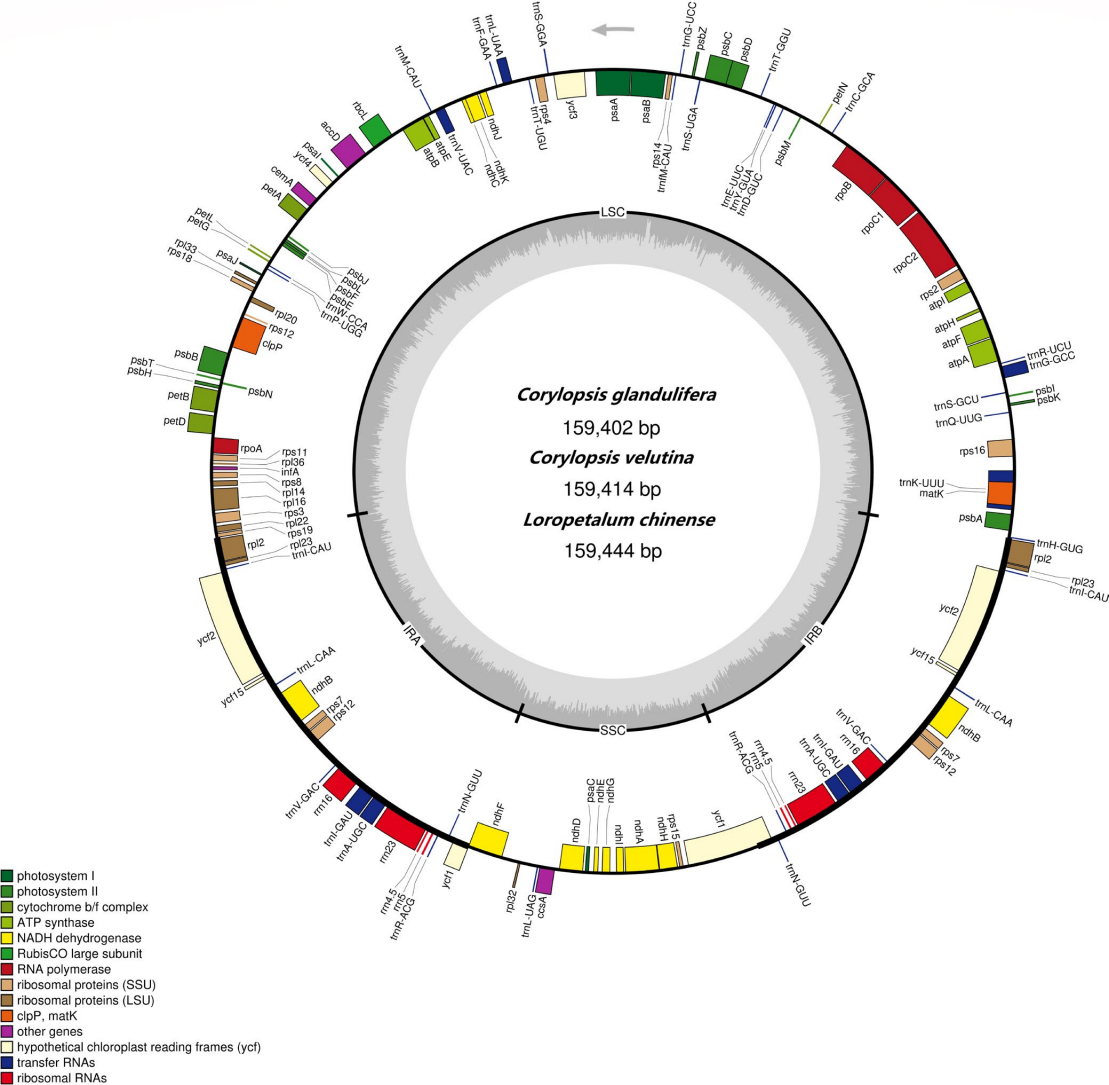
The chloroplast genome maps of *Corylopsis glandulifera*, *Corylopsis velutina*, and *Loropetalum chinense*. Genes on the inside of the circle are transcribed clockwise and those on the outside are transcribed counter‐clockwise. The darker gray inner circle corresponds to the GC content, whereas the lighter gray indicates the AT content. Different colors represent different functional genes

The chloroplast genomes of *C*. *glandulifera* (accession no. MZ642354), *C*. *velutina* (accession no. MZ823391), and *L*. *chinense* (accession no. MZ642355) have been submitted to GenBank at the NCBI. The genome sizes ranged from 159,402 bp (*C*. *glandulifera*) to 159,444 bp (*L*. *chinense*). These chloroplast genomes had a circular assembly and exhibited a typical quadripartite structure containing an LSC region (88,134–88,160 bp) and an SSC region (18,702–18,770 bp) separated by IR regions (26,257–26,083 bp) (Figure 1, Table 1). The overall GC contents of the three chloroplast genomes were almost identical (37.97%–38.03%) (Table 1) and the GC contents of the LSC and SSC regions were lower than those of the IR regions (Table 2).

**TABLE 1:** Summary of the complete chloroplast genomes of the three Hamamelidaceae species

Genome features	*Corylopsis velutina*	*Corylopsis glandulifera*	*Loropetalum chinense*
Total length (bp)	159,414	159,402	159,444
LSC length (bp)	88,146	88,134	88,160
SSC length (bp)	18,702	18,702	18,770
IRa length (bp)	26,283	26,283	26,257
IRb length (bp)	26,283	26,283	26,257
Genes	133	132	132
Protein‐coding genes (CDS)	87	87	85
tRNA genes	37	37	38
rRNA genes	8	8	8
GC%	38.03	38.03	37.97

**TABLE 2:** Base composition of the complete chloroplast genomes of the three Hamamelidaceae species

Species	Region	A (%)	T (U) (%)	C (%)	G (%)	AT (%)	GC (%)
*Corylopsis velutina*	LSC	31.26	32.60	18.61	17.53	63.86	36.14
	SSC	33.65	33.67	17.11	15.57	67.32	32.68
	IR	28.44	28.44	21.55	21.55	56.88	43.10
	Total	30.61	31.36	19.40	18.63	61.97	38.03
*Corylopsis glandulifera*	LSC	31.26	32.59	18.61	17.53	63.85	36.14
	SSC	33.69	33.67	17.11	15.54	67.36	32.64
	IR	28.45	28.45	21.55	21.55	56.90	43.10
	Total	30.62	31.35	19.41	18.62	61.97	38.03
*Loropetalum chinense*	LSC	31.29	32.65	18.58	17.49	63.94	36.07
	SSC	33.62	33.70	17.19	15.49	67.32	32.67
	IR	28.46	28.46	21.53	21.53	56.92	43.06
	Total	30.63	31.39	19.39	18.59	62.02	37.97

The chloroplast genomes of *C*. *glandulifera* and *L*. *chinense* encoded 132 genes, including 87 protein‐coding genes, 37 tRNA genes, and 8 rRNA genes in *C*. *glandulifera* and 85 protein‐coding genes, 38 tRNA genes, 8 rRNA genes, and 1 pseudogene (*ycf*1) in *L*. *chinense*. A total of 133 distinct genes were annotated in the *C*. *velutina* chloroplast genome, including 87 protein‐coding genes, 37 tRNA genes, 8 rRNA genes, and 1 pseudogene (*ycf*1) (Table 1). After removing the duplicates, 80 protein‐coding genes, 30 tRNA genes, and 4 rRNA genes remained in *C*. *glandulifera* and *C*. *velutina*, while 79 protein‐coding genes, 29 tRNA genes, and 4 rRNA genes remained in *L*. *chinense*. The LSC region comprised 62 protein‐coding genes and 22 tRNA genes, while the SSC region comprised 11 protein‐coding and 1 tRNA gene of the three chloroplast genomes (Figure 1, Figures S1 and S2). Twenty genes contained introns: 17 genes (*ndhA*, *ndhB*, *petB*, *petD*, *atpF*, *rpl16*, *rpl2*, *rps16*, *rpoC1*, *trnA*‐*UGC*, *trnG*‐*GCC*, *trnG*‐*UCC*, *trnI*‐*GAU*, *trnK*‐*UUU*, *trnL*‐*UAA*, *trnV*‐*UAC*, and *trnE*‐*UUC*) contained 1 intron, while 3 genes (*rps12*, *clpP*, and *ycf3*) possessed two introns (Table 3). *trnK*‐*UUU* featured the longest intron (2,441–2,457 bp) and the shortest intron was found in *trnL*‐*UAA* (521–516 bp) (Table 4). Notably, *rps12* was considered a trans‐spliced gene separated by two introns, with 1 exon in the LSC region and the other 2 in the IR regions (Figure 1).

**TABLE 3:** Lists of genomic genes for *Corylopsis velutina*, *Corylopsis glandulifera*, and *Loropetalum chinense*

Function	*C. velutina* Genes	*C. glandulifera* Genes	*L. chinense* Genes
Photosystem I	*psaA*,*psaB*,*psaC*,*psaI*,*psaJ*		
Photosystem II	*psbA*,*psbB*,*psbC*,*psbD*,*psbE*,*psbF*,*psbH*,*psbI*,*psbJ*,*psbK*,*psbL*,*psbM*,*psbN*,*psbT*,*psbZ*		
NADH dehydrogenase	ndhA*,ndhB*(2),*ndhC*,*ndhD*,*ndhE*,*ndhF*,*ndhG*,*ndhH*,*ndhI*,*ndhJ*,*ndhK*		
Cytochrome b/f complex	*petA*,petB*,petD*,*petG*,*petL*,*petN*		
ATP synthase	*atpA*,*atpB*,*atpE*,atpF*,*atpH*,*atpI*		
Rubisco	*rbcL*		
Large subunit ribosomal proteins	*rpl14*,rpl16*,rpl2*(2),*rpl20*,*rpl22*,*rpl23*(2),*rpl32*,*rpl33*,*rpl36*		
Small subunit ribosomal proteins	*rps11*,rps12**(2),*rps14*,*rps15*,rps16*,*rps18*,*rps19*,*rps2*,*rps3*,*rps4*,*rps7*(2),*rps8*		
RNA polymerase	*rpoA*,*rpoB*,rpoC1*,*rpoC2*		
Ribosomal RNAs	*rrn16(2)*,*rrn23*(2),*rrn4*.*5*(2),*rrn5*(2)		
Transfer RNAs	trnA‐UGC*(2),trnC‐GCA,trnD‐GUC,trnE‐UUC,trnF‐GAA,trnG‐GCC*,trnG‐UCC,trnH‐GUG,trnI‐CAU(2),trnI‐GAU*(2),trnK‐UUU*,trnL‐CAA(2),trnL‐UAA*,trnL‐UAG,trnM‐CAU,trnN‐GUU(2),trnP‐UGG,trnQ‐UUG,trnR‐ACG(2),trnR‐UCU,trnS‐GCU,trnS‐GGA,trnS‐UGA,trnT‐GGU,trnT‐UGU,trnV‐GAC(2),trnV‐UAC*,trnW‐CCA,trnY‐GUA,trnfM‐CAU	trnA‐UGC*(2),trnC‐GCA,trnD‐GUC,trnE‐UUC,trnF‐GAA,trnG‐GCC,trnG‐UCC*,trnH‐GUG,trnI‐CAU(2),trnI‐GAU*(2),trnK‐UUU*,trnL‐CAA(2),trnL‐UAA*,trnL‐UAG,trnM‐CAU,trnN‐GUU(2),trnP‐UGG,trnQ‐UUG,trnR‐ACG(2),trnR‐UCU,trnS‐GCU,trnS‐GGA,trnS‐UGA,trnT‐GGU,trnT‐UGU,trnV‐GAC(2),trnV‐UAC*,trnW‐CCA,trnY‐GUA,trnfM‐CAU	trnA‐UGC*(2),trnC‐GCA,trnD‐GUC,trnE‐UUC,trnE‐UUC*,trnF‐GAA,trnG‐GCC*,trnG‐UCC,trnH‐GUG,trnI‐CAU(2),trnI‐GAU*(2),trnK‐UUU*,trnL‐CAA(2),trnL‐UAA*,trnL‐UAG,trnM‐CAU,trnN‐GUU(2),trnP‐UGG,trnQ‐UUG,trnR‐ACG(2),trnR‐UCU,trnS‐GCU(2),trnS‐UGA,trnT‐GGU,trnT‐UGU,trnV‐GAC(2),trnV‐UAC*,trnW‐CCA,trnY‐GUA,trnfM‐CAU
Other	*matK*,clpP**,*cemA*,*accD*,*ccsA*,*infA*		
Unknown function	#*ycf1*,*ycf1*,*ycf15*(2),*ycf2*(2),ycf3**,*ycf4*	*ycf1*(2),*ycf15*(2),*ycf2*(2),ycf3**,*ycf4*	#*ycf1*,*ycf1*,*ycf2*(2),ycf3**,*ycf4*

Note

*, Gene with one intron; **, Gene with two introns; #, Pseudogene; (2): Gene with two copies.

**TABLE 4:** Characteristics and sizes of the intron and exon genes from the three Hamamelidaceae species

Species	Gene	Exon I (bp)	Intron I (bp)	Exon II (bp)	Intron II (bp)	Exon III (bp)
*Corylopsis velutina*	*trnK‐UUU*	37	2,441	37		
	*rps16*	39	850	225		
	*trnG‐GCC*	34	688	48		
	*atpF*	159	712	411		
	*rpoC1*	435	735	1,632		
	*ycf3*	126	746	228	741	153
	*trnL‐UAA*	37	515	50		
	*trnV‐UAC*	39	574	37		
	*rps12*	114	–	232	538	26
	*clpP*	69	635	291	812	228
	*petB*	6	744	651		
	*petD*	9	690	474		
	*rpl16*	9	1,001	402		
	*rpl2*	393	653	435		
	*ndhB*	777	682	756		
	*rps12*	232	–	26	538	114
	*trnI‐GAU*	42	939	30		
	*trnA‐UGC*	38	842	35		
	*ndhA*	552	1,073	540		
	*trnA‐UGC*	38	842	35		
	*trnI‐GAU*	42	939	30		
	*ndhB*	777	682	756		
	*rpl2*	393	653	435		
*Corylopsis glandulifera*	*trnK‐UUU*	37	2,443	35		
	*rps16*	39	851	225		
	*trnG‐UCC*	34	687	48		
	*atpF*	159	712	411		
	*rpoC1*	435	735	1,632		
	*ycf3*	126	746	228	741	153
	*trnL‐UAA*	37	516	50		
	*trnV‐UAC*	39	574	37		
	*rps12*	114	–	232	538	26
	*clpP*	69	631	291	812	228
	*petB*	6	744	651		
	*petD*	9	690	474		
	*rpl16*	9	1,001	402		
	*rpl2*	393	653	435		
	*ndhB*	777	682	756		
	*rps12*	232	–	26	538	114
	*trnI‐GAU*	42	939	30		
	*trnA‐UGC*	38	842	35		
	*ndhA*	552	1,073	540		
	*trnA‐UGC*	38	842	35		
	*trnI‐GAU*	42	939	30		
	*ndhB*	777	682	756		
	*rpl2*	393	653	435		
*Loropetalum chinense*	*trnK‐UUU*	37	2,457	35		
	*rps16*	42	853	225		
	*trnG‐GCC*	24	699	48		
	*atpF*	159	697	426		
	*rpoC1*	427	752	1,625		
	*ycf3*	126	742	228	757	156
	*trnL‐UAA*	37	512	50		
	*trnV‐UAC*	39	574	32		
	*rps12*	114	–	232	538	26
	*clpP*	69	644	291	836	228
	*petB*	6	781	654		
	*petD*	9	690	474		
	*rpl16*	9	1,005	402		
	*rpl2*	393	653	435		
	*ndhB*	777	682	756		
	*rps12*	232	–	26	538	114
	*trnI‐GAU*	42	890	35		
	*trnA‐UGC*	38	842	35		
	*ndhA*	552	1,042	540		
	*trnA‐UGC*	38	842	35		
	*trnE‐UUC*	33	939	41		
	*trnI‐GAU*	42	890	35		
	*ndhB*	777	682	756		
	*rpl2*	393	653	435		

### 3.2 Codon usage analysis

**FIGURE 2:**
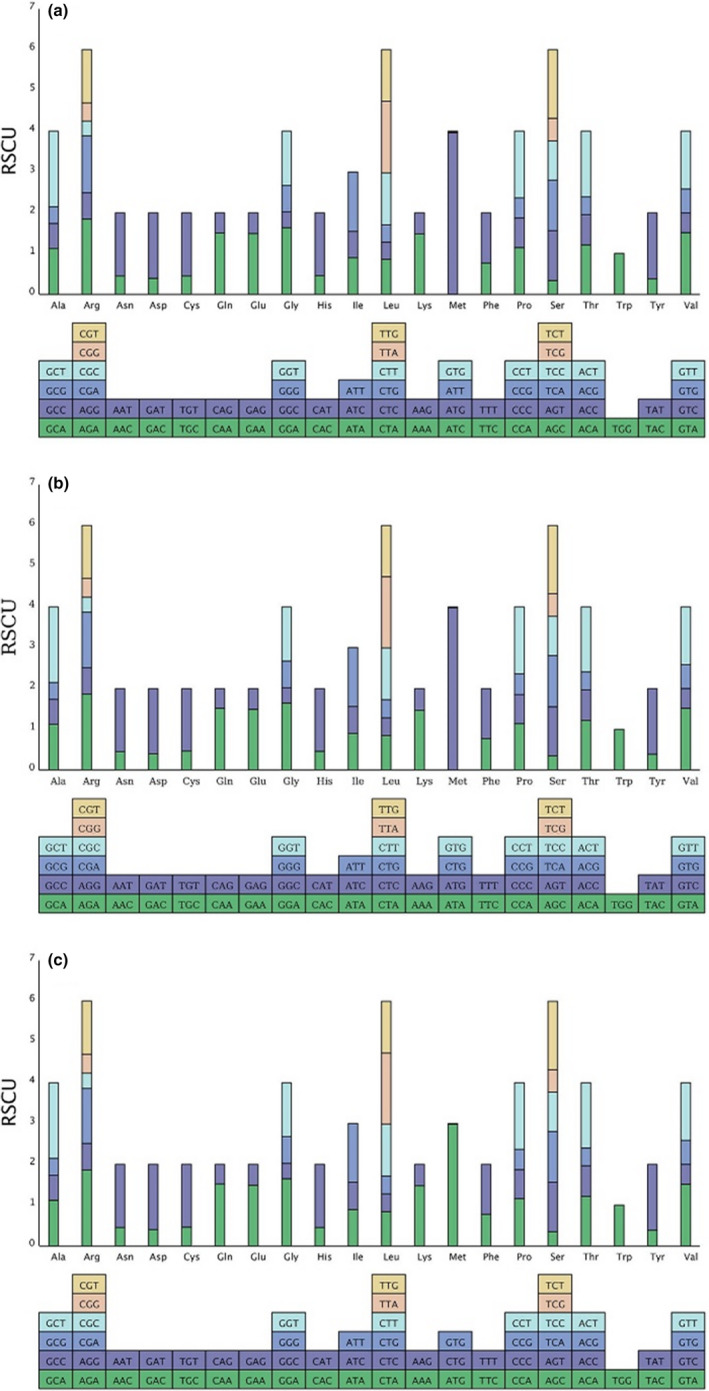
Codon content of 20 amino acids and stop codons in the protein‐coding genes of the chloroplast genomes of the three Hamamelidaceae species. (a) *Loropetalum chinense*; (b) *Corylopsis glandulifera*; (c) *Corylopsis velutina*

Analyzing codon usage is essential to evaluate the evolution of the chloroplast genome (Chi et al., 2020; Sun et al., 2021). RSCU values were computed for the *C*. *glandulifera*, *C*. *velutina*, and *L*. *chinense* chloroplast genomes based on the protein‐coding sequences. Figure 2 shows the codon content of 20 amino acids and stop codons in all protein‐coding genes of the chloroplast genomes of the three species. The coding regions of *C*. *glandulifera*, *C*. *velutina*, and *L*. *chinense* were composed of 26,797, 26,574, and 26,415 codons, respectively. The most prevalent amino acid was leucine (2,811 codons in *C*. *glandulifera*, 2,781 codons in *C*. *velutina*, and 2,764 codons in *L*. *chinense*), while the rarest one was cysteine (319 codons in *C*. *glandulifera*, 317 codons in *C*. *velutina*, and 318 codons in *L*. *chinense*). Codons with no preference value were set to 1.00. Codons for arginine, leucine, and serine were the most abundant (RSCU = 6), while those for methionine and tryptophan were the least abundant (RSCU = 1) (Figure 2), indicating no codon bias for these two amino acids. In addition, nearly all the A/U‐ending codons had RSCU values >1, whereas the C/G‐ending codons had RSCU values <1 (Table S2), indicating that most of the amino acids tended to use A/U‐ending codons rather than C/G‐ending codons.

### 3.3 Repeat sequence analysis

**FIGURE 3:**
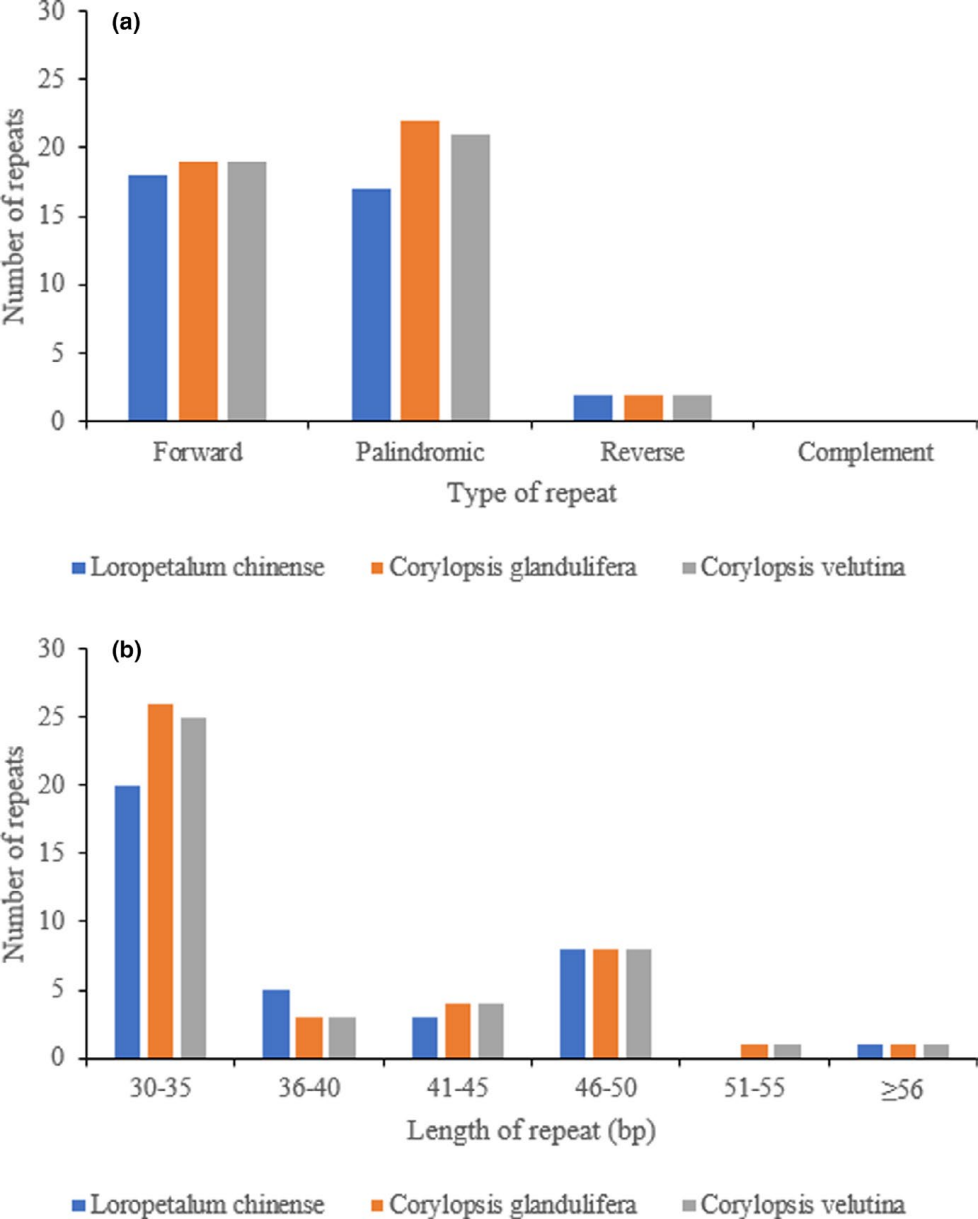
Analysis of repeated sequences in the three Hamamelidaceae chloroplast genomes. (a) Frequency of repeat types; (b) Frequency of repeat sequences by length

Structures longer than 30 bp are known as long repeats (Asaf et al., 2018), and there are four types of long repeats, such as forward, palindromic, reverse, and complement repeats. In this study, three types of repeated sequences (forward, reverse, and palindromic) were detected in the chloroplast genomes of the three Hamamelidaceae species. In detail, there were 43 (19 forward, 22 palindromes, and 2 reverse), 42 (19 forward, 21 palindrome, and 2 reverse), and 37 (18 forward, 17 palindrome, and 2 reverse) long repeats in *C*. *glandulifera*, *C*. *velutina*, and *L*. *chinense*, respectively (Figure 3a). The lengths of the dispersed repeats were 30–35 bp (Figure 3b). Most of the long repeats were located in the *ycf* gene and the intergenic spacer (IGS) (Table S3). The types and content of the long repeats were similar in species from the same genus.

### 3.4 SSR analysis

**FIGURE 4:**
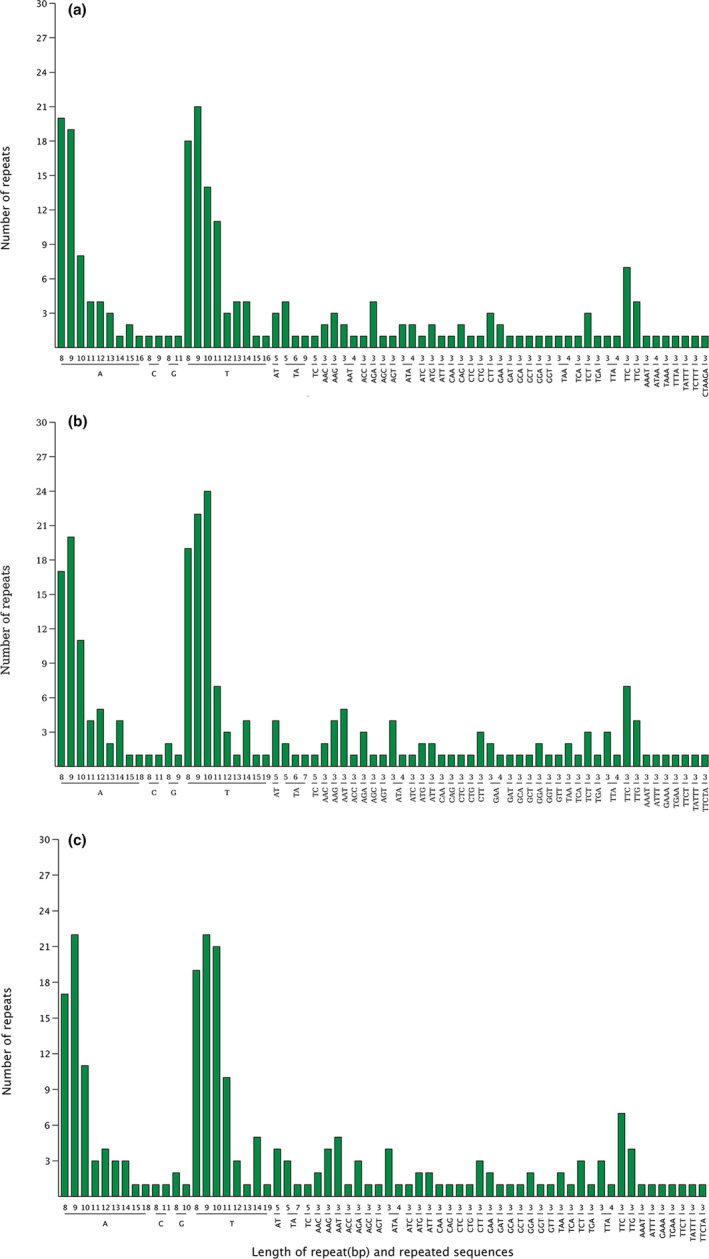
Frequency of SSRs in the different repeat class types. (a) *Loropetalum chinense*; (b) *Corylopsis glandulifera*; (c) *Corylopsis velutina*

Six types of SSRs were detected, including mononucleotides, dinucleotides, trinucleotides, tetranucleotides, pentanucleotides, and hexanucleotides with a total of 175–178 SSRs in the three species. The majority of the SSRs were located in intergenic regions. Most of the SSRs were located in the LSC regions rather than in the SSC or IR regions (Table S4). There were 143–152 mononucleotides, 9–10 dinucleotides, 58–66 trinucleotides, 4–5 tetranucleotides, 2 pentanucleotides, and 0–1 hexanucleotide (only in *L*. *chinense*). Among these SSRs, mononucleotide repeats were the most abundant, while pentanucleotide repeats numbered the least. Most mononucleotides and dinucleotides were composed of A and T (Figure 4).

### 3.5 Comparative genomic analysis

**FIGURE 5:**
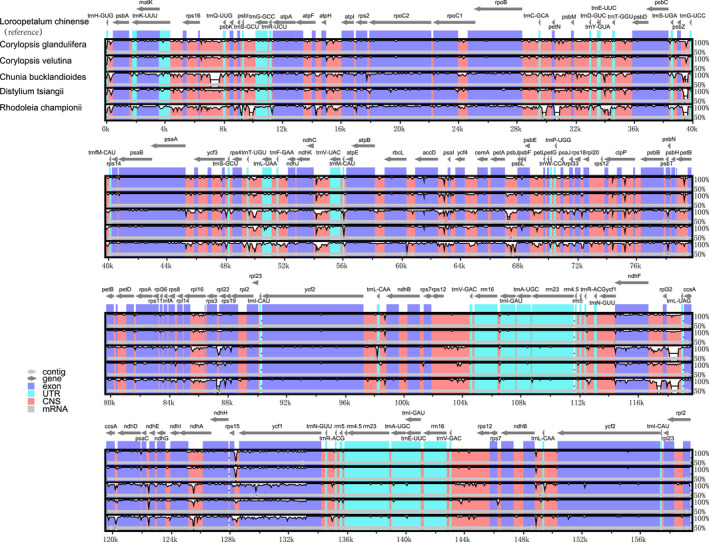
Complete chloroplast genome alignments of six Hamamelidaceae species using the mVISTA program, with the chloroplast genome of *Loropetalum chinense* as a reference. The horizontal axis indicates the coordinates within the chloroplast genome. The vertical scale indicates the percent identity within 50–100%. Annotated genes are displayed along the top

To investigate genomic divergence, the percentage of sequence identity was calculated for six species of Hamamelidaceae using the mVISTA program with *L*. *chinense* as the reference. The results showed that the similarity among the six species was high and the variability in the IR regions was less than that in the LSC and SSC regions. Furthermore, the chloroplast genomes were more highly variable in their noncoding regions than in their coding regions and this is consistent with the pattern found in most angiosperms (Yang et al., 2020) (Figure 5).

**FIGURE 6:**
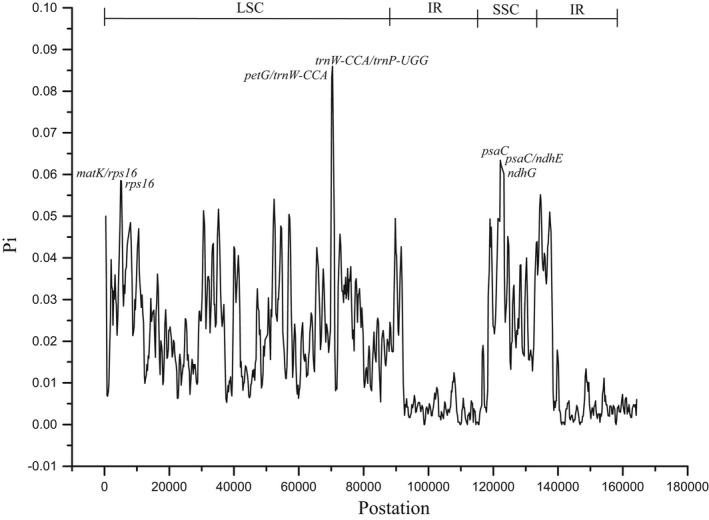
Nucleotide diversity (*Pi*) values among the six Hamamelidaceae species. X‐axis: the position in the genome; Y‐axis: *Pi* value. *Pi*, polymorphism information

The chloroplast genome contains many variable nucleotides, which can be used to resolve closely related species or genera as valuable DNA barcoding (Liu et al., 2019; Xiong et al., 2020). In this study, variable loci were identified in the six species, with polymorphism information (*Pi*) values ranging from 0.0000 to 0.08600. According to the sliding‐window analysis, the variation in the LSC region was the greatest, followed by the SSC region, and the IR regions were the least variable (Figure 6). Seven of these loci, that is, *matK*‐*rps16* (0.05856), *rps16* (0.05844), *petG*‐*trnW* (0.08333), *trnW*‐*trnP* (0.08600), *psaC* (0.06344), *psaC*‐*ndhE* (0.06233), and *ndhG* (0.06011), showed high values (>0.055). Among them, 4 fragments were distributed in the LSC region and 3 in the SSC region (Figure 6).

### 3.6 IR contraction and expansion

**FIGURE 7:**
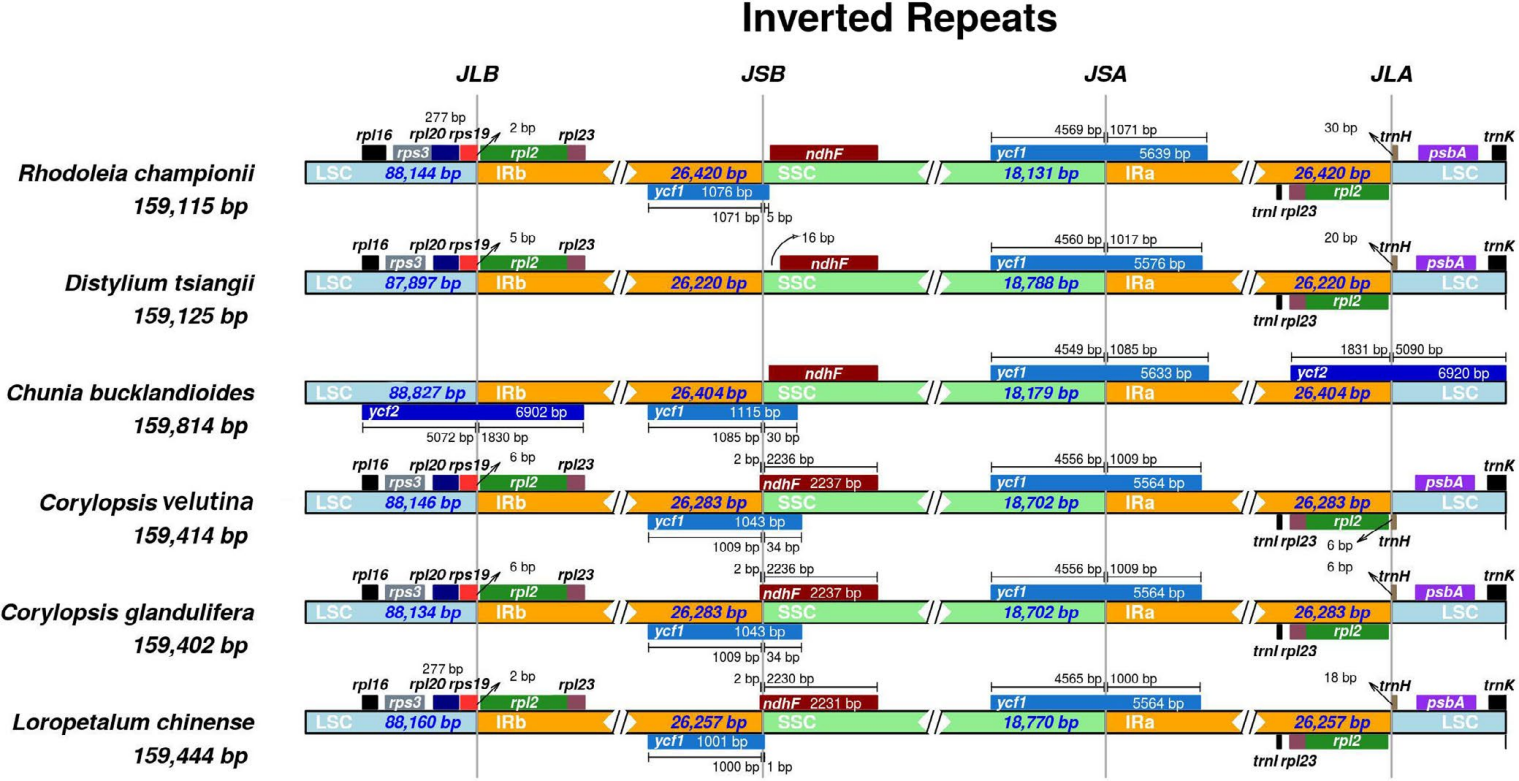
Comparison of the borders of the large single‐copy (LSC), small single‐copy (SSC), and inverted repeat (IR) regions among the six Hamamelidaceae chloroplast genomes. Genes are denoted by colored boxes. The gaps between the genes and the boundaries are indicated by the base lengths (bp)

Figure 7 shows the comparisons of the IR/LSC and IR/SSC boundaries among the chloroplast genomes of the six Hamamelidaceae species. The length of the chloroplast genome of *Chunia bucklandioides* was the longest (159,814 bp), while that of *Rhodoleia championii* was the shortest (159,115 bp) among the six species. The genes *rps19*, *ndhF*, *ycf1*, and *trnH* were located at the LSC/IRb, IRb/SSC, SSC/IRa, and IRa/LSC boundaries, respectively. *rps19* crossed the LSC/IRb boundary, with 2–6 bp within the IRb region, while *trnH* crossed the IRa/LSC boundary, with 6–30 bp within the IRb region, except in *Chunia bucklandioides*. *NdhF* was located away from the IRb/SSC boundary in the SSC regions of *Rhodoleia championii*, *Distylium tsiangii*, and *Chunia bucklandioides* but crossed the IRb/SSC boundary with 2 bp within the IRb region in *C*. *velutina*, *C*. *glandulifera*, and *L*. *chinense*. Notably, *ycf1* crossed the SSC/IRa boundary, with 1,000–1,085 bp within the IRa region in all six species.

### 3.7 Phylogenetic analysis

**FIGURE 8:**
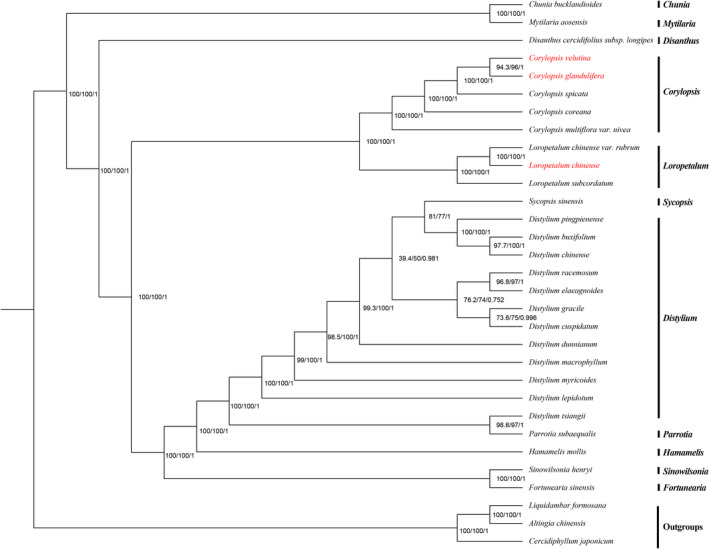
Bayesian inference (BI) and maximum likelihood (ML) phylogenetic trees were constructed using the general time‐reversible (GTR)+F+I+G4 model based on the chloroplast genomes of 31 species. Numbers are support values for ML‐SH‐Alrt, ML‐UFBoot, and BI‐PP (SH‐aLRT/UFBoot/PP). The species investigated in this study are colored in red

**FIGURE 9:**
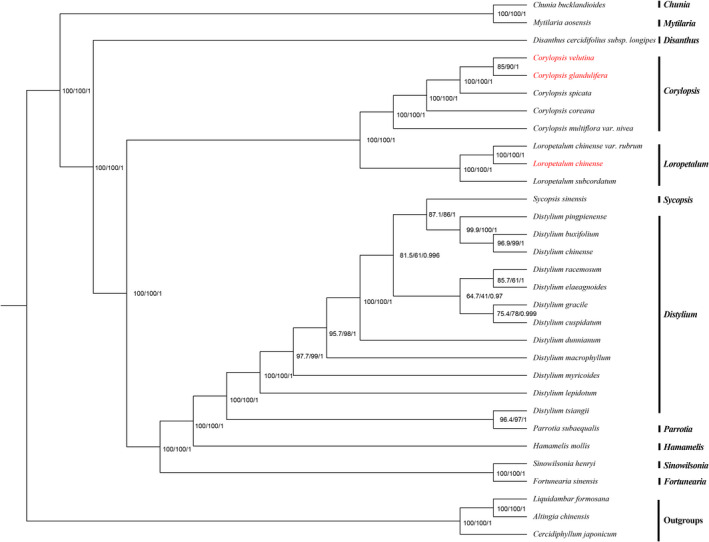
Bayesian inference (BI) and maximum likelihood (ML) phylogenetic trees were constructed using the general time‐reversible (GTR)+F+I+G4 model based on the LSC regions. Numbers on the branches are support values for ML‐SH‐Alrt, ML‐UFBoot, and BI‐PP (SH‐aLRT/UFBoot/PP). The species investigated in this study are colored in red

**FIGURE 10:**
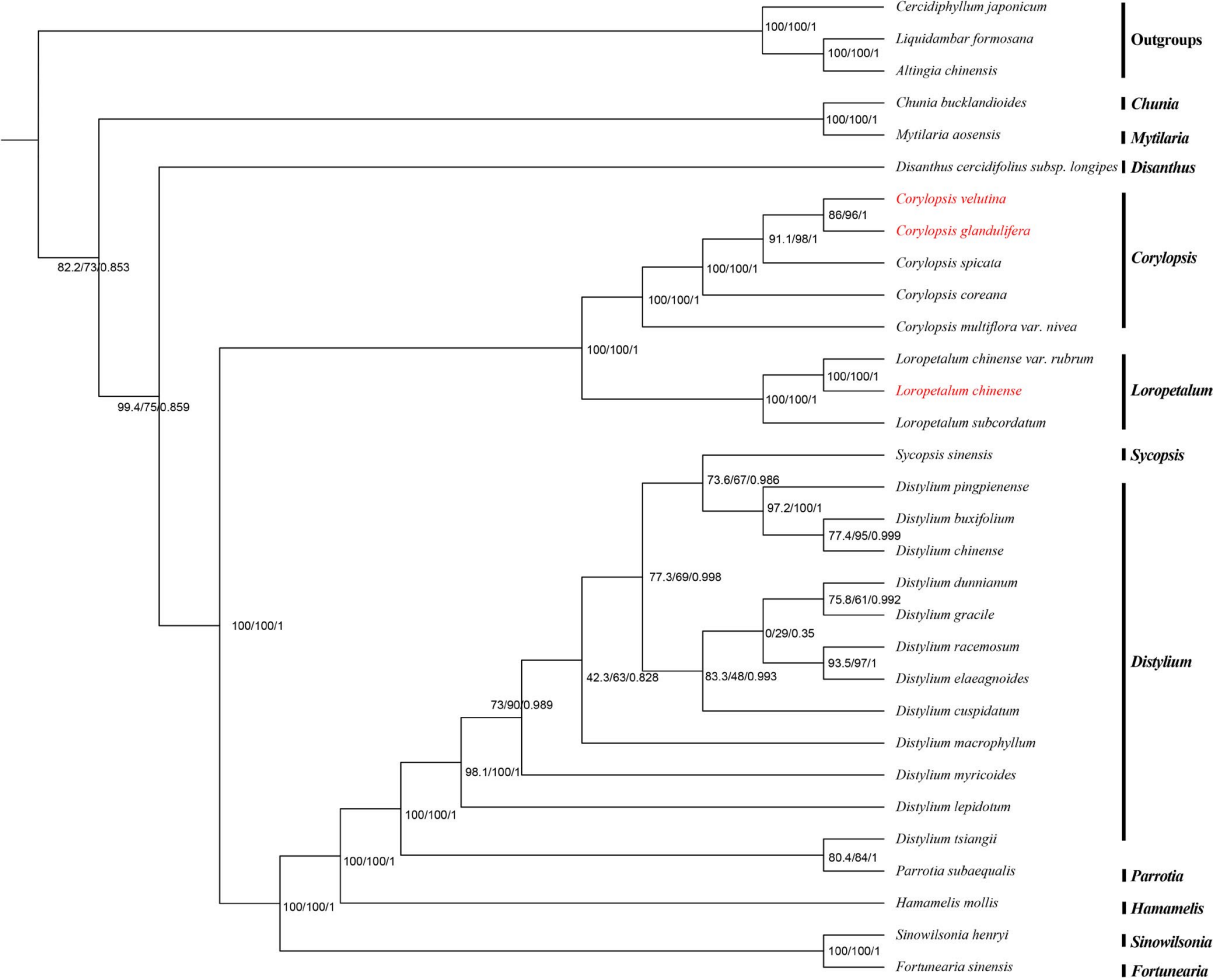
Bayesian inference (BI) and maximum likelihood (ML) phylogenetic trees were constructed using the general time‐reversible (GTR)+F+G4 model based on the SSC regions. Numbers on the branches are support values for ML‐SH‐Alrt, ML‐UFBoot, and BI‐PP (SH‐aLRT/UFBoot/PP). The species investigated in this study are colored in red

The chloroplast genome sequences observed provide essential data with which to further elucidate and understand the phylogenetic relationships among Hamamelidaceae species. The two phylogenetic analyses (ML and BI) revealed nearly identical topologies based on the complete chloroplast genomes, LSC regions, and SSC regions (the complete chloroplast genome was completely consistent with the LSC region), and all of the nodes in the phylogenetic trees had high bootstrap support values except *Distylium* (some *Distylium* species) and *Sycopsis* Oliv. (*Sycopsis sinensis* Oliver) (Figures 8, 9, 10). Hamamelidaceae species gathered on a large branch and species in the same genus were clustered together to a certain degree. The Hamamelidaceae branch was divided into two clades with *Chunia* and *Mytilaria* related to other 9 genera. *Disanthus* was related to other 8 genera in which *Corylopsis* and *Loropetalum* were found to be sister to other 6 genera (*Sinowilsonia* Hemsl., *Fortunearia*, *Sycopsis*, *Distylium*, *Parrotia*, and *Hamamelis*). In addition, *Corylopsis* and *Loropetalum* were sister genera to each other. However, ML and BI analyses revealed incongruent topologies based on the IR regions. Moreover, some of the nodes had very low bootstrap support values (Figure S3), indicating that the IR regions were not suitable for identification or phylogenetic analysis.

## 4 DISCUSSION

The chloroplast genome provides valuable information for species identification, as well as population genetics, phylogenetic, and genetic engineering studies (Daniell et al., 2016; Luo et al., 2021; Wu et al., 2021). In this study, the complete chloroplast genomes of three Hamamelidaceae species were sequenced using Illumina high‐throughput sequencing technology. The results showed that the three Hamamelidaceae species had classical chloroplast structure (He et al., 2017; Mader et al., 2018; Xu et al., 2017; Yang, Hu, et al., 2018; Yang, Zhao, et al., 2018) and the GC content was lower than the AT content. This was generally the same as seen in other angiosperm chloroplast genomes (Asaf et al., 2018; Raubeson et al., 2007). The results also showed that the GC content in the IR regions was the highest, which may be due to the presence of a large number of rRNA in the IR regions. GC skewness is considered a dominant factor in codon bias. Several studies have indicated that high AT content is the main reason for synonymous codons ending in A/U (Clegg et al., 1994; Shimda & Sugiuro, 1991), which may be related to natural selection and mutation during evolution (Liu et al., 2019). In addition, SSRs are usually composed of a higher number of A + T bases than G + C bases (Hu et al., 2017; Kuang et al., 2011; Simeone et al., 2018; Yang, Hu, et al., 2018; Yang, Zhao, et al., 2018), which is consistent with our observations, and this may also be related to the high AT content in the nucleotide composition.

The lengths of the exons and introns in genes are important information in chloroplast genomes. Genes are interrupted by introns in major groups of organisms (Fan et al., 2021). One‐intron genes vary among species, while *clpP*, *rps12*, and *ycf3* are two‐intron genes (Wu et al., 2020; Zhang, Gao, et al., 2020; Zhang, Wang, et al., 2020). This finding is consistent with our observations. *ClpP* protease encoded by the *clpP* gene widely exists in mitochondria and chloroplasts of prokaryotes and eukaryotes, where it plays a vital role in regulating protein metabolism (Chen et al., 2014; Zhang et al., 2014). The *rps12* gene is a trans‐spliced gene with the 5′ end located in the LSC region and duplicated 3′ ends located in the IR regions (Guo et al., 2018). In addition, *ycf3* is related to photosynthesis (Boudreau et al., 1997; Naver et al., 2001). Consequently, detecting the *clpP* and *ycf3* genes will contribute to further investigation of the chloroplasts in Hamamelidaceae.

The LSC and SSC regions are usually variable, while expansion and contraction are noted in the highly conserved IR regions (Asaf, 2017), which may be a critical factor underlying the size variation in the chloroplast genomes (Daniell et al., 2016; Kolodner et al., 1976). The difference in the size of the chloroplast genomes among the six Hamamelidaceae species compared in this study was not significant, which could be due to their similar expansion and contraction in the IR regions (such as *rps19*, *ndhF*, *ycf1*, and *trnH* located at the LSC/IRb, IRb/SSC, SSC/IRa, and IRa/LSC boundaries, respectively) except *C*. *bucklandioides*. The longest chloroplast genome among the six Hamamelidaceae species was observed in *C*. *bucklandioides*, which may be associated with the size expansion of *ycf2* in the IR regions. Expansion or contraction of the IR regions in these species is supposed to be related to gene retention or loss, and we suggest that gene‐loss events would have occurred during the evolution of this family and differentiation of the species.

The nucleotide diversity analysis also demonstrated that the IR regions contained fewer variable loci than the SC regions (LSC and SSC regions). Moreover, genes with *Pi* values > 0.055 were mainly located in the SC regions. Chloroplast genomes have a copy‐dependent repair mechanism to ensure consistency and stability of the two IR regions in sequence, which enhances the stability and conservation of the genome (Khakhlova & Bock, 2006; Perry & Wolfe, 2002). This could explain why the IR regions contain less sequence divergence than the LSC or SSC regions (Shaw et al., 2007). None of the intron‐containing genes (*ndhA*, *ndhB*, *petB*, *petD*, *atpF*, *rpl16*, *rpl2*, *rpoC1*, *trnA*‐*UGC*, *trnG*‐*GCC*, *trnG*‐*UCC*, *trnI*‐*GAU*, *trnK*‐*UUU*, *trnL*‐*UAA*, *trnV*‐*UAC*, *trnE*‐*UUC*, *rps12*, *clpP*, and *ycf3*) had a *Pi* value >0.055, except *rps16*, suggesting that intron‐containing genes are more highly conserved than exon‐containing genes only in the chloroplast genome. In other words, higher variability was found in exon‐containing genes, which provides more valuable information for species evolution.

The mVISTA analysis also showed that the variability in the IR regions was less than that in the LSC and SSC regions and that more variable sites were located in noncoding regions than in coding regions, which could be utilized for the development of new molecular markers for species identification and taxonomic studies in Hamamelidaceae. These variations were observed in the *ndhF*, *accD*, and *ycf1* genes and intergenic regions, such as *trnH*‐*psbA*, *rps16*‐*trnQ*, *atpH*‐*atpI*, *petN*‐*psbM*, *trnT*‐*psbD*, *psbZ*‐*trnG*, *ndhC*‐*trnV*, *accD*‐*psaI*, *petA*‐*psbJ*, *rps18*‐*rpl20*, *rps15*‐*ycf1*, and *trnL*‐*ndhB*. Among these divergence hotspot regions, *trnH*‐*psbA* has already been selected as a suitable barcode for plants (Hollingsworth et al., 2009; Yang et al., 2017), as well as *rbcL*‐*accD* (Shaw et al., 2014), *ndhF* (Chen et al.,^2021^; Yang et al., 2017; Yang, Hu, et al., 2018; Yang, Zhao, et al., 2018), *ycf1* (Dong et al., 2015), *accD* (Li et al., 2018), *rps16* (Chen et al.,^2021^), *rps16*‐*trnQ* (Liu et al., 2016), and *petA*‐*psbJ* (Katarzyna et al., 2018; Liu et al., 2016; Wang, 2010). Further research is necessary to determine whether the remaining divergence hotspot regions could be used as candidate DNA barcodes or to assess the taxonomic evolution and phylogenetics of Hamamelidaceae.

Chloroplast genome data are valuable for analyzing species definitions because organelle‐based “barcodes” can be established for some species and then applied to reveal the phylogenetic relationships among species (Fan et al., 2021; Yang et al., 2013). Moreover, with the continuous development of next‐generation sequencing technology, particularly the application of second‐generation sequencing technology, chloroplast genome sequencing has become simpler. Thus, more studies have used complete chloroplast genome sequences to evaluate the phylogenetic relationships among angiosperms. In this study, almost all published complete chloroplast genome sequence data of Hamamelidaceae were used to construct the phylogenetic relationships by ML and BI analyses. The two phylogenetic analyses (ML and BI) revealed congruent topologies based on the complete chloroplast genomes and LSC regions, while the results of the SSC regions were slightly different in the two datasets. This may be because the LSC region accounts for a large part of the complete chloroplast genome and varies highly. However, the IR region was not suitable for identification or the phylogenetic analysis possibly because it is highly conserved. The outgroups, *Altingia chinensis*, *Liquidambar formosana*, and *Cercidiphyllum japonicum*, clustered into a monophyletic clade and were sister to Hamamelidaceae. According to previous molecular studies on Saxifragales, Altingiaceae (*Altingia*, *Liquidambar*), and Cercidiphyllaceae (*Cercidiphyllum*), Hamamelidaceae originated successively in the evolutionary history of angiosperms, and the three groups are paraphyletic (Dong et al., 2013, 2018; Soltis et al., 2013; Tarullo et al., 2021; Xiang et al., 2019). Alternatively, a different relationship of these paraphyletic groups was inferred from the morphological and molecular data, with an earlier divergence time for *Cercidiphyllum* than for *Liquidambar* (Magallon, 2007). Our results are slightly different from previous studies, possibly due to sample limitations. Nevertheless, we still support the establishment of Altingiaceae (APG IV, 2016). The results show that the Hamamelidaceae species investigated in this study were divided into two clades and the species in the same genus were clustered together to a certain degree. Among them, *Chunia bucklandioides* and *Mytilaria laosensis* (Xiang et al., 2019) were the first to diverge in Hamamelidaceae, indicating the relatively high genetic divergence between these two species and others, followed by *Disanthus cercidifolius* subsp. *longipes*, which were early‐diverging taxa in Hamamelidaceae. Interestingly, these three genera are monotypic. *Corylopsis* and *Loropetalum* formed a monophyletic group, while *Fortunearia* was closely related to *Sinowilsonia* and they are monotypic genera endemic to China (Chen et al., 2020; Jiang et al., 2020; Ye et al., 2020). The sister relationships of the three clades in Hamamelidaceae, such as *Chunia* + Mytilaria, *Disanthus*, and *Corylopsis* + Loropetalum, are consistent with previous reports (Bobrov et al., 2020; Tarullo et al., 2021; Xiang et al., 2019), while *Distylium* is not monophyletic. Moreover, some *Distylium* support values in the phylogenetic trees were low, particularly in the ML analysis. Although Dong et al. (2021) conducted a phylogenetic analysis on *Distylium* species, the support values were not high, possibly due to the close affinity within the *Distylium* genus and therefore the classification or circumscription would be difficult within *Distylium*. There are still unsolved enigmas in the phylogeny of Hamamelidaceae. This group is disjunctly distributed across Western, Southern, Eastern, and Southeast Asia; North, Central, and South America; Eastern Africa; and Northeastern Australia (Bobrov et al., 2020; Tarullo et al., 2021). The diversity in Hamamelidaceae is not fully understood, and extinct and extant new species are still being reported (Averyanov et al., 2017; Haynes et al., 2020; Huang et al., 2017). Therefore, the morphological and molecular evidence may not be complete due to sampling difficulties. Conversely, the unresolved mysteries in Hamamelidaceae may lead to more follow‐up studies. To fully understand the phylogeny of Hamamelidaceae species, studies on more genera and more genes need to be conducted in the future. Nevertheless, the phylogenetic trees constructed in this study provide a valuable resource for investigating the classification, phylogeny, and evolutionary history of Hamamelidaceae.

## 5 CONCLUSION

In this study, the complete chloroplast genomes of three Hamamelidaceae species were determined and the basic structures, conservation, and variability in these sequences were revealed. The IR regions were more conserved than the LSC or SSC region, while the noncoding regions contained more variability than the gene coding regions. SSRs and divergent hotspot regions could be used to develop molecular markers for population genetic and phylogenetic studies. The complete chloroplast genomes, LSC regions, and SSC regions were used to establish good phylogenetic relationships and solve the relationships between and within genera, while the IR region was not suitable for identification or phylogenetic analysis. Notably, the relationship within the genus *Distylium* has not been well resolved. More studies on the relationship within this genus are needed to fully understand the phylogeny of Hamamelidaceae species. The results of this study provide a valuable reference for further studies on species identification, determination of evolutionary relationships, and the development of genetic resources within Hamamelidaceae.

## CONFLICT OF INTEREST

The authors declare no conflicts of interest regarding publication of this paper.

## AUTHOR CONTRIBUTIONS

**NingJie Wang:** Conceptualization (lead); Data curation (lead); Methodology (equal); Resources (lead); Software (lead); Visualization (lead); Writing – original draft (lead). **ShuiFei Chen:** Conceptualization (equal); Data curation (equal); Funding acquisition (equal); Writing – original draft (equal). **Lei Xie:** Conceptualization (equal); Writing – review & editing (equal). **Lu Wang:** Methodology (equal); Resources (equal). **YueYao Feng:** Resources (equal). **Ting Lv:** Formal analysis (equal); Visualization (equal). **YanMing Fang:** Funding acquisition (lead); Writing – review & editing (lead). **Hui Ding:** Funding acquisition (equal); Writing – review & editing (equal).

## Supporting information

Appendix S1

## Data Availability

The original sequencing data have been submitted to the NCBI database and received GenBank accession numbers MZ642354 (*C*. *glandulifera*), MZ823391 (*C*. *velutina*), and MZ642355 (*L*. *chinense*). The data used to support the findings of this study are included in Appendix S1.
