# Supplementary material for: The complete chloroplast genomes of three Hamamelidaceae species: Comparative and phylogenetic analyses
Source: Ecol Evol. 2022 Feb 16;12(2):e8637. doi: 10.1002/ece3.8637 (PMC8848467; doi:10.1002/ece3.8637)
Supplement: Supplementary file 1 — Appendix S1 [file ECE3-12-e8637-s001.docx]

**Appendix**

1. Fastp v0.20.0 (https://github.com/OpenGene/fastp) was used to filter the original data. The filtering criteria were as follows:

(1) Truncate the sequencing linker and primer sequence in the reads;

(2) Filter out the reads whose average quality value is < Q5;

(3) Filter out n reads whose number is > 5;

The high-quality reads obtained after the above series of quality control steps were the clean data.

2. SPAdes v3.10.1 (Bankevich et al., 2012) was used for genome assembly; kmer used 55, 87, and 121 respectively, and the assembly did not depend on the reference genome. However, due to the characteristics of second-generation sequencing, genomic repeats, genome-specific structure, and other reasons, the complete circular genome sequence could not be directly obtained by one-time splicing. Other strategies were used to obtain the complete circular genome sequence. The assembly process was divided into the following 7 steps:

Step 1: The seed sequence of the chloroplast genome was obtained by assembling the cpDNA sequences with SPAdes v3.10.1.

Step 2: kmer iterative extend seed if the result of step 2 was a contig, the result was determined to be a pseudo genome sequence, and step 6 was performed directly.

Step 3: Connect the contig sequence obtained in step 2 with SSPACE v2.0 (Acemel et al., 2016) to obtain the scaffolds.

Step 4: Use Gapfiller v2.1.1 (Boetzer et al., 2012) to make up the gaps for the scaffold sequences obtained in step 3.

Step 5: If a gap still existed after these operations, design primers, conduct PCR sequencing, and reassemble until the complete pseudo genome sequence was obtained.

Step 6: The sequencing reads were aligned to the pseudo genome to correct the genome.

Step7: According to the structure of the chloroplast, the corrected pseudo genome was rearranged to obtain the complete chloroplast circular genome sequence.


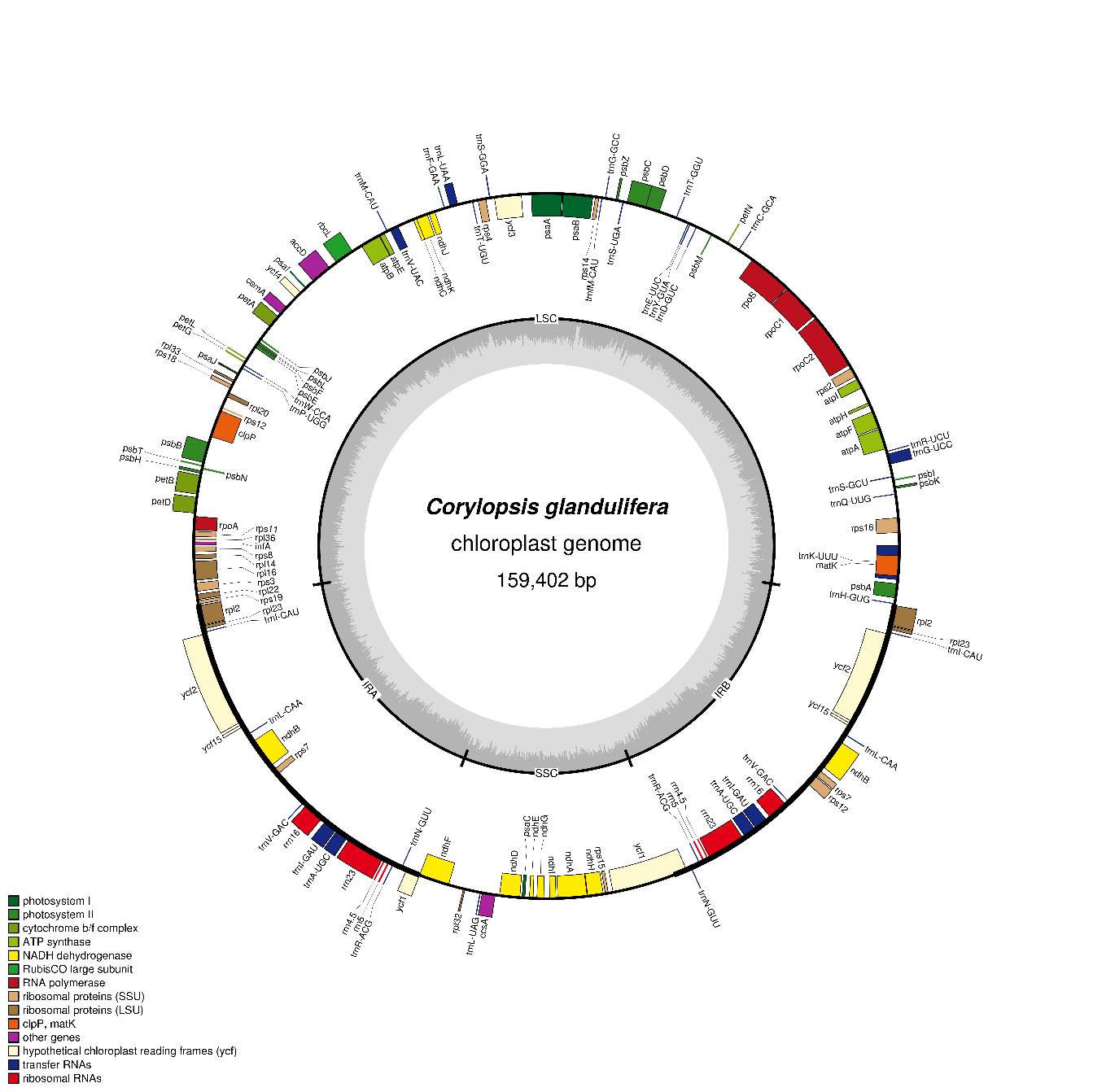


Figure S1 Chloroplast genome map of *C*. *glandulifera*.


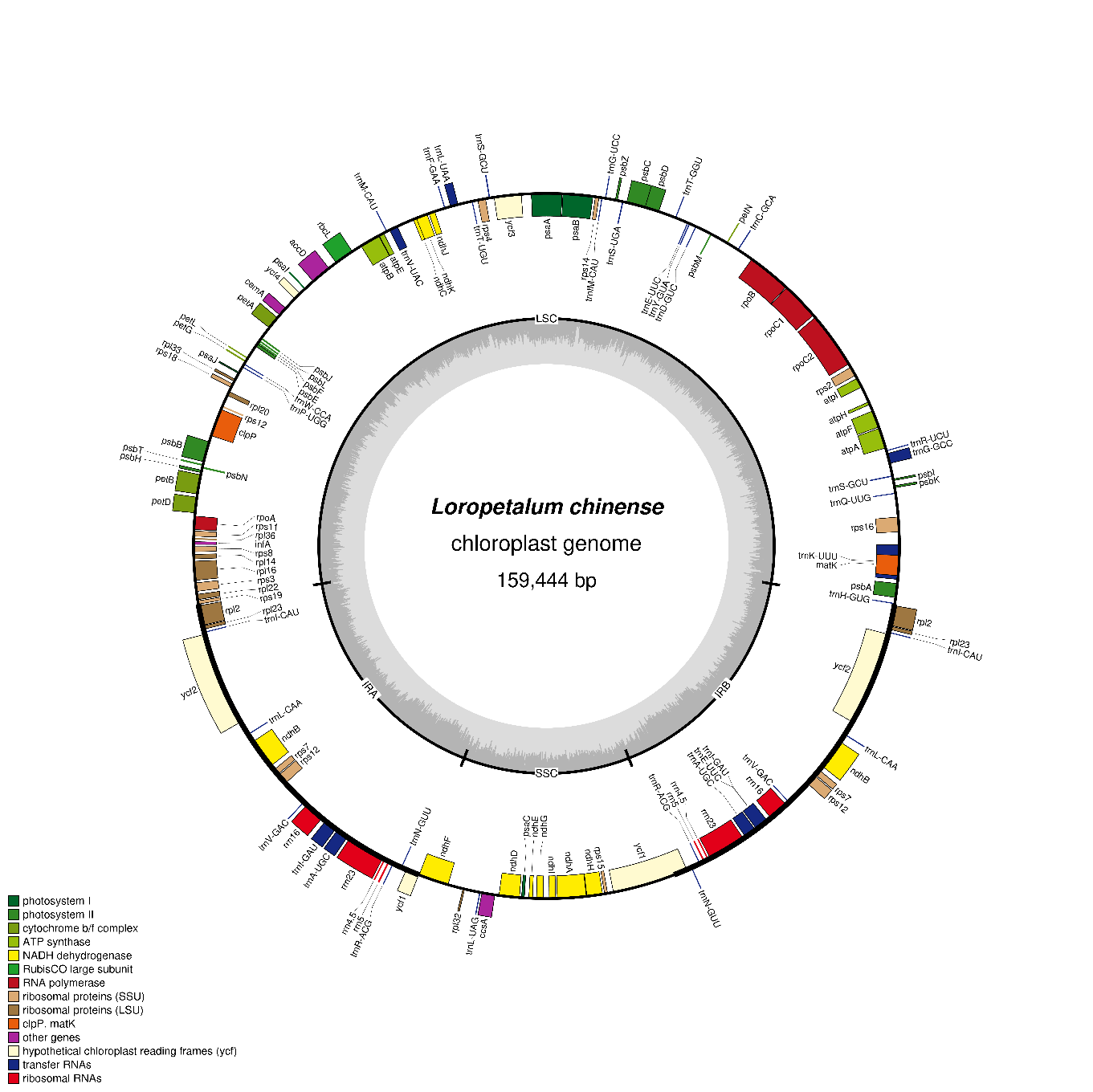


Figure S2 Chloroplast genome map of *L*. *chinense*.


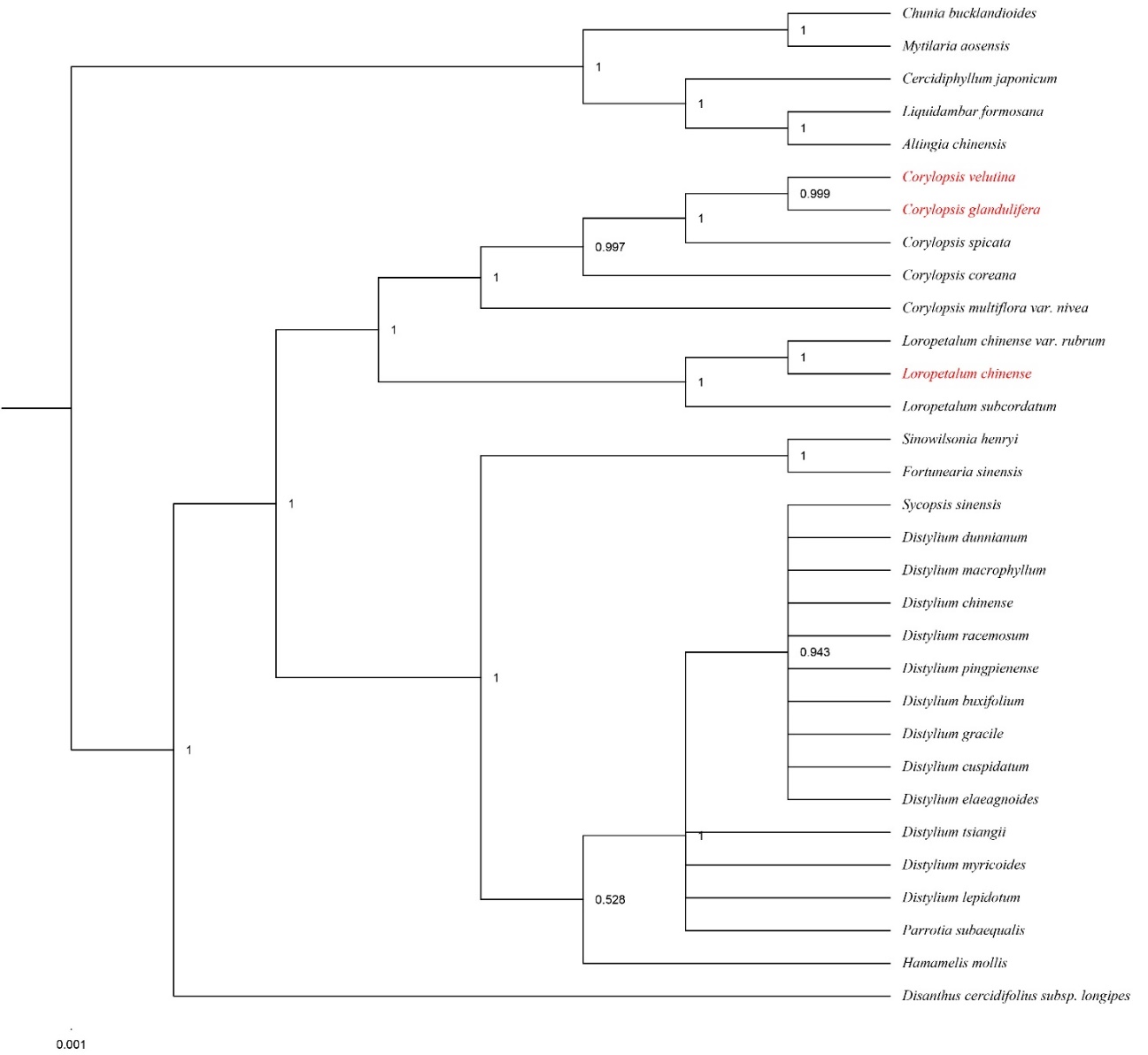


Figure S3a. The Bayesian inference (BI) phylogenetic tree was constructed using the general time-reversible (GTR)+F+I model based on the IR regions. Numbers on the branches are BI-PP (PP) support values. The species investigated in this study are colored in red.


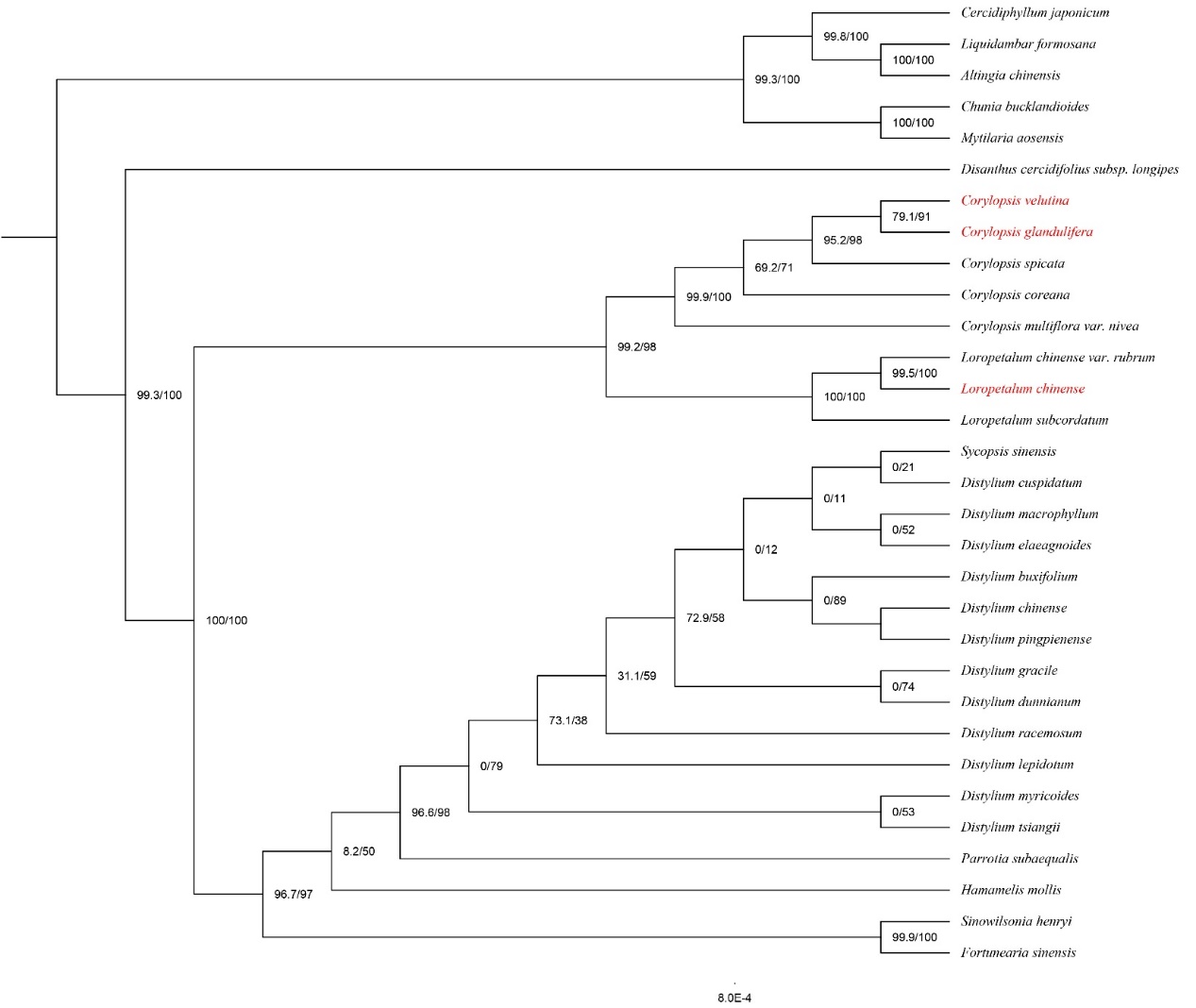


Figure S3b. Maximum likelihood (ML) phylogenetic tree was constructed using general time-reversible (GTR)+F+I model based on the IR regions. Numbers on the branches are support values for ML-SH-Alrt and ML-UFBoot (SH-aLRT/UFBoot). The species investigated in this study are colored in red.

Table S1 GenBank accession numbers of the complete chloroplast genome sequences of 28 species obtained from the NCBI.

| Species | Accession number |
| --- | --- |
| *Chunia bucklandioides* | NC_041163 |
| *Corylopsis coreana* | NC_040141 |
| *Corylopsis multiflora* var. *nivea* | MW043717 |
| *Corylopsis spicata* | MK942341 |
| *Disanthus cercidifolius* subsp. *longipes* | NC_050371 |
| *Distylium buxifolium* | MW248115 |
| *Distylium chinense* | MW248112 |
| *Distylium cuspidatum* | MW248117 |
| *Distylium dunnianum* | MW248109 |
| *Distylium elaeagnoides* | MW248120 |
| *Distylium gracile* | MW248116 |
| *Distylium lepidotum* | MW248119 |
| *Distylium macrophyllum* | MW248111 |
| *Distylium myricoides* | MW248110 |
| *Distylium pingpienense* | MW248114 |
| *Distylium racemosum* | MW248113 |
| *Distylium tsiangii* | MN711651 |
| *Fortunearia sinensis* | MN496061 |
| *Hamamelis mollis* | NC_037881 |
| *Loropetalum chinense* var. *rubrum* | MW368385 |
| *Loropetalum subcordatum* | NC_037694 |
| *Mytilaria laosensis* | NC_048997 |
| *Parrotia subaequalis* | NC_037243 |
| *Sinowilsonia henryi* | MF497447 |
| *Sycopsis sinensis* | MN496080 |
| *Cercidiphyllum japonicum* | MG605672 |
| *Liquidambar formosana* | KC588388 |
| *Altingia chinensis* | MT193687 |

Table S2 The codon number and relative synonymous codon usage (RSCU) in the complete chloroplast genomes of *C*. *glandulifera*, *C*. *velutina*, and *L*. *chinense*.

| Species | Amino Acid | Symbol | Codon | No. | RSCU |
| --- | --- | --- | --- | --- | --- |
| *C. glandulifera* | * | Ter | UAA | 43 | 1.4829 |
|  | * | Ter | UAG | 25 | 0.8622 |
|  | * | Ter | UGA | 19 | 0.6552 |
|  | A | Ala | GCA | 404 | 1.126 |
|  | A | Ala | GCC | 219 | 0.6104 |
|  | A | Ala | GCG | 149 | 0.4152 |
|  | A | Ala | GCU | 663 | 1.848 |
|  | C | Cys | UGC | 75 | 0.4702 |
|  | C | Cys | UGU | 244 | 1.5298 |
|  | D | Asp | GAC | 226 | 0.4024 |
|  | D | Asp | GAU | 897 | 1.5976 |
|  | E | Glu | GAA | 1051 | 1.4908 |
|  | E | Glu | GAG | 359 | 0.5092 |
|  | F | Phe | UUC | 574 | 0.7716 |
|  | F | Phe | UUU | 914 | 1.2284 |
|  | G | Gly | GGA | 746 | 1.644 |
|  | G | Gly | GGC | 171 | 0.3768 |
|  | G | Gly | GGG | 298 | 0.6568 |
|  | G | Gly | GGU | 600 | 1.3224 |
|  | H | His | CAC | 155 | 0.4662 |
|  | H | His | CAU | 510 | 1.5338 |
|  | I | Ile | AUA | 679 | 0.8979 |
|  | I | Ile | AUC | 503 | 0.6651 |
|  | I | Ile | AUU | 1087 | 1.4373 |
|  | K | Lys | AAA | 1019 | 1.4652 |
|  | K | Lys | AAG | 372 | 0.5348 |
|  | L | Leu | CUA | 396 | 0.8454 |
|  | L | Leu | CUC | 206 | 0.4398 |
|  | L | Leu | CUG | 208 | 0.444 |
|  | L | Leu | CUU | 594 | 1.2678 |
|  | L | Leu | UUA | 818 | 1.746 |
|  | L | Leu | UUG | 589 | 1.257 |
|  | M | Met | AUA | 1 | 0.006 |
|  | M | Met | AUG | 648 | 3.9756 |
|  | M | Met | CUG | 1 | 0.006 |
|  | M | Met | GUG | 2 | 0.0124 |
|  | N | Asn | AAC | 296 | 0.4536 |
|  | N | Asn | AAU | 1009 | 1.5464 |
|  | P | Pro | CCA | 321 | 1.1424 |
|  | P | Pro | CCC | 200 | 0.7116 |
|  | P | Pro | CCG | 142 | 0.5052 |
|  | P | Pro | CCU | 461 | 1.6404 |
|  | Q | Gln | CAA | 714 | 1.5176 |
|  | Q | Gln | CAG | 227 | 0.4824 |
|  | R | Arg | AGA | 502 | 1.866 |
|  | R | Arg | AGG | 174 | 0.6468 |
|  | R | Arg | CGA | 366 | 1.3608 |
|  | R | Arg | CGC | 99 | 0.3678 |
|  | R | Arg | CGG | 125 | 0.4644 |
|  | R | Arg | CGU | 348 | 1.2936 |
|  | S | Ser | AGC | 122 | 0.3516 |
|  | S | Ser | AGU | 419 | 1.2072 |
|  | S | Ser | UCA | 434 | 1.251 |
|  | S | Ser | UCC | 335 | 0.9654 |
|  | S | Ser | UCG | 194 | 0.5592 |
|  | S | Ser | UCU | 578 | 1.6656 |
|  | T | Thr | ACA | 420 | 1.2184 |
|  | T | Thr | ACC | 259 | 0.7512 |
|  | T | Thr | ACG | 151 | 0.438 |
|  | T | Thr | ACU | 549 | 1.5924 |
|  | V | Val | GUA | 546 | 1.5156 |
|  | V | Val | GUC | 176 | 0.4884 |
|  | V | Val | GUG | 211 | 0.5856 |
|  | V | Val | GUU | 508 | 1.41 |
|  | W | Trp | UGG | 469 | 1 |
|  | Y | Tyr | UAC | 190 | 0.389 |
|  | Y | Tyr | UAU | 787 | 1.611 |
|  |  |  |  | 26797 |  |
| *C. velutina* | * | Ter | UAA | 44 | 1.5171 |
|  | * | Ter | UAG | 24 | 0.8277 |
|  | * | Ter | UGA | 19 | 0.6552 |
|  | A | Ala | GCA | 402 | 1.122 |
|  | A | Ala | GCC | 220 | 0.614 |
|  | A | Ala | GCG | 147 | 0.4104 |
|  | A | Ala | GCU | 664 | 1.8536 |
|  | C | Cys | UGC | 73 | 0.4606 |
|  | C | Cys | UGU | 244 | 1.5394 |
|  | D | Asp | GAC | 224 | 0.4004 |
|  | D | Asp | GAU | 895 | 1.5996 |
|  | E | Glu | GAA | 1031 | 1.491 |
|  | E | Glu | GAG | 352 | 0.509 |
|  | F | Phe | UUC | 563 | 0.7718 |
|  | F | Phe | UUU | 896 | 1.2282 |
|  | G | Gly | GGA | 745 | 1.6464 |
|  | G | Gly | GGC | 171 | 0.378 |
|  | G | Gly | GGG | 297 | 0.6564 |
|  | G | Gly | GGU | 597 | 1.3192 |
|  | H | His | CAC | 149 | 0.4542 |
|  | H | His | CAU | 507 | 1.5458 |
|  | I | Ile | AUA | 674 | 0.8976 |
|  | I | Ile | AUC | 500 | 0.6657 |
|  | I | Ile | AUU | 1079 | 1.4367 |
|  | K | Lys | AAA | 1014 | 1.476 |
|  | K | Lys | AAG | 360 | 0.524 |
|  | L | Leu | CUA | 391 | 0.8436 |
|  | L | Leu | CUC | 200 | 0.4314 |
|  | L | Leu | CUG | 204 | 0.4404 |
|  | L | Leu | CUU | 589 | 1.2708 |
|  | L | Leu | UUA | 809 | 1.7454 |
|  | L | Leu | UUG | 588 | 1.2684 |
|  | M | Met | AUG | 639 | 2.9859 |
|  | M | Met | CUG | 1 | 0.0048 |
|  | M | Met | GUG | 2 | 0.0093 |
|  | N | Asn | AAC | 293 | 0.4542 |
|  | N | Asn | AAU | 997 | 1.5458 |
|  | P | Pro | CCA | 325 | 1.1564 |
|  | P | Pro | CCC | 201 | 0.7152 |
|  | P | Pro | CCG | 139 | 0.4948 |
|  | P | Pro | CCU | 459 | 1.6336 |
|  | Q | Gln | CAA | 716 | 1.5186 |
|  | Q | Gln | CAG | 227 | 0.4814 |
|  | R | Arg | AGA | 501 | 1.8636 |
|  | R | Arg | AGG | 174 | 0.6474 |
|  | R | Arg | CGA | 364 | 1.3542 |
|  | R | Arg | CGC | 100 | 0.372 |
|  | R | Arg | CGG | 125 | 0.465 |
|  | R | Arg | CGU | 349 | 1.2984 |
|  | S | Ser | AGC | 120 | 0.3492 |
|  | S | Ser | AGU | 416 | 1.2108 |
|  | S | Ser | UCA | 428 | 1.2462 |
|  | S | Ser | UCC | 331 | 0.9636 |
|  | S | Ser | UCG | 190 | 0.5532 |
|  | S | Ser | UCU | 576 | 1.677 |
|  | T | Thr | ACA | 415 | 1.2172 |
|  | T | Thr | ACC | 255 | 0.748 |
|  | T | Thr | ACG | 148 | 0.434 |
|  | T | Thr | ACU | 546 | 1.6012 |
|  | V | Val | GUA | 543 | 1.5156 |
|  | V | Val | GUC | 174 | 0.4856 |
|  | V | Val | GUG | 210 | 0.586 |
|  | V | Val | GUU | 506 | 1.4124 |
|  | W | Trp | UGG | 465 | 1 |
|  | Y | Tyr | UAC | 187 | 0.3868 |
|  | Y | Tyr | UAU | 780 | 1.6132 |
|  |  |  |  | 26574 |  |
| *L. chinense* | * | Ter | UAA | 44 | 1.5528 |
|  | * | Ter | UAG | 23 | 0.8118 |
|  | * | Ter | UGA | 18 | 0.6354 |
|  | A | Ala | GCA | 399 | 1.1256 |
|  | A | Ala | GCC | 218 | 0.6148 |
|  | A | Ala | GCG | 144 | 0.4064 |
|  | A | Ala | GCU | 657 | 1.8532 |
|  | C | Cys | UGC | 72 | 0.4528 |
|  | C | Cys | UGU | 246 | 1.5472 |
|  | D | Asp | GAC | 221 | 0.3978 |
|  | D | Asp | GAU | 890 | 1.6022 |
|  | E | Glu | GAA | 1025 | 1.493 |
|  | E | Glu | GAG | 348 | 0.507 |
|  | F | Phe | UUC | 556 | 0.7668 |
|  | F | Phe | UUU | 894 | 1.2332 |
|  | G | Gly | GGA | 740 | 1.638 |
|  | G | Gly | GGC | 174 | 0.3852 |
|  | G | Gly | GGG | 293 | 0.6484 |
|  | G | Gly | GGU | 600 | 1.328 |
|  | H | His | CAC | 149 | 0.4592 |
|  | H | His | CAU | 500 | 1.5408 |
|  | I | Ile | AUA | 672 | 0.8985 |
|  | I | Ile | AUC | 486 | 0.6498 |
|  | I | Ile | AUU | 1086 | 1.452 |
|  | K | Lys | AAA | 1014 | 1.4814 |
|  | K | Lys | AAG | 355 | 0.5186 |
|  | L | Leu | CUA | 396 | 0.8598 |
|  | L | Leu | CUC | 193 | 0.4188 |
|  | L | Leu | CUG | 196 | 0.4254 |
|  | L | Leu | CUU | 589 | 1.2786 |
|  | L | Leu | UUA | 807 | 1.752 |
|  | L | Leu | UUG | 583 | 1.2654 |
|  | M | Met | AUC | 1 | 0.0064 |
|  | M | Met | AUG | 632 | 3.956 |
|  | M | Met | AUU | 4 | 0.0252 |
|  | M | Met | GUG | 2 | 0.0124 |
|  | N | Asn | AAC | 287 | 0.4526 |
|  | N | Asn | AAU | 981 | 1.5474 |
|  | P | Pro | CCA | 318 | 1.148 |
|  | P | Pro | CCC | 201 | 0.7256 |
|  | P | Pro | CCG | 136 | 0.4908 |
|  | P | Pro | CCU | 453 | 1.6352 |
|  | Q | Gln | CAA | 700 | 1.5054 |
|  | Q | Gln | CAG | 230 | 0.4946 |
|  | R | Arg | AGA | 497 | 1.845 |
|  | R | Arg | AGG | 175 | 0.6498 |
|  | R | Arg | CGA | 375 | 1.3926 |
|  | R | Arg | CGC | 97 | 0.36 |
|  | R | Arg | CGG | 120 | 0.4458 |
|  | R | Arg | CGU | 352 | 1.3068 |
|  | S | Ser | AGC | 117 | 0.3414 |
|  | S | Ser | AGU | 418 | 1.2192 |
|  | S | Ser | UCA | 425 | 1.2396 |
|  | S | Ser | UCC | 330 | 0.9624 |
|  | S | Ser | UCG | 192 | 0.5598 |
|  | S | Ser | UCU | 575 | 1.677 |
|  | T | Thr | ACA | 414 | 1.2132 |
|  | T | Thr | ACC | 254 | 0.7444 |
|  | T | Thr | ACG | 148 | 0.4336 |
|  | T | Thr | ACU | 549 | 1.6088 |
|  | V | Val | GUA | 541 | 1.5144 |
|  | V | Val | GUC | 173 | 0.4844 |
|  | V | Val | GUG | 211 | 0.5908 |
|  | V | Val | GUU | 504 | 1.4108 |
|  | W | Trp | UGG | 458 | 1 |
|  | Y | Tyr | UAC | 182 | 0.3804 |
|  | Y | Tyr | UAU | 775 | 1.6196 |
|  |  |  |  | 26415 |  |

Notes:

Amino acid: single letter abbreviation for amino acids; Symbol: three-letter abbreviation for the amino acids; *: stop codon; No.: number of codons; RSCU: codon preference.

Table S3 Long repeats in the complete chloroplast genomes of *C*. *glandulifera*, *C*. *velutina*, and *L*. *chinense*.

| Species | ID | Repeat I Start | Repeat II Start | Type | Size(bp) | Distance | Gene | Region |
| --- | --- | --- | --- | --- | --- | --- | --- | --- |
| *C. glandulifera* | 1 | 87341 | 87409 | F | 51 | -3 | rpl22;rpl22 | LSC;LSC |
|  | 2 | 92916 | 92937 | F | 47 | -3 | ycf2;ycf2 | IRb;IRb |
|  | 3 | 92916 | 154554 | P | 47 | -3 | ycf2;ycf2 | IRb;IRa |
|  | 4 | 92937 | 154575 | P | 47 | -3 | ycf2;ycf2 | IRb;IRa |
|  | 5 | 154554 | 154575 | F | 47 | -3 | ycf2;ycf2 | IRa;IRa |
|  | 6 | 95369 | 95387 | F | 46 | -1 | ycf2;ycf2 | IRb;IRb |
|  | 7 | 95369 | 152105 | P | 46 | -1 | ycf2;ycf2 | IRb;IRa |
|  | 8 | 95387 | 152123 | P | 46 | -1 | ycf2;ycf2 | IRb;IRa |
|  | 9 | 152105 | 152123 | F | 46 | -1 | ycf2;ycf2 | IRa;IRa |
|  | 10 | 102703 | 125128 | F | 42 | 0 | IGS;ndhA | IRb;SSC |
|  | 11 | 125128 | 144793 | P | 42 | 0 | ndhA;IGS | SSC;IRa |
|  | 12 | 47021 | 125127 | F | 42 | -3 | ycf3;ndhA | LSC;SSC |
|  | 13 | 42058 | 44282 | F | 41 | -3 | psaB;psaA | LSC;LSC |
|  | 14 | 47024 | 102705 | F | 39 | -2 | ycf3;IGS | LSC;IRb |
|  | 15 | 47024 | 144794 | P | 39 | -2 | ycf3;IGS | LSC;IRa |
|  | 16 | 87356 | 87424 | F | 36 | -2 | rpl22;rpl22 | LSC;LSC |
|  | 17 | 87327 | 87395 | F | 35 | -3 | IGS;rpl22 | LSC;LSC |
|  | 18 | 154569 | 154590 | F | 32 | -1 | ycf2;ycf2 | IRa;IRa |
|  | 19 | 9192 | 38460 | F | 32 | -3 | trnS-GCU;trnS-UGA | LSC;LSC |
|  | 20 | 108221 | 108259 | F | 31 | -2 | trnA-UGC;trnA-UGC | IRb;IRb |
|  | 21 | 108221 | 139248 | P | 31 | -2 | trnA-UGC;trnA-UGC | IRb;IRa |
|  | 22 | 108259 | 139286 | P | 31 | -2 | trnA-UGC;trnA-UGC | IRb;IRa |
|  | 23 | 121890 | 121893 | P | 31 | -2 | IGS | SSC;SSC |
|  | 24 | 139248 | 139286 | F | 31 | -2 | trnA-UGC;trnA-UGC | IRa;IRa |
|  | 25 | 95366 | 95402 | F | 31 | -3 | ycf2;ycf2 | IRb;IRb |
|  | 26 | 95366 | 152105 | P | 31 | -3 | ycf2;ycf2 | IRb;IRa |
|  | 27 | 95402 | 152141 | P | 31 | -3 | ycf2;ycf2 | IRb;IRa |
|  | 28 | 152102 | 152138 | F | 31 | -3 | ycf2;ycf2 | IRa;IRa |
|  | 29 | 7536 | 7540 | R | 31 | -3 | IGS | LSC;LSC |
|  | 30 | 9194 | 48177 | P | 30 | 0 | trnS-GCU;trnS-GGA | LSC;LSC |
|  | 31 | 33904 | 33904 | P | 30 | 0 | IGS | LSC;LSC |
|  | 32 | 49932 | 49934 | P | 30 | -1 | IGS | LSC;LSC |
|  | 33 | 114009 | 114009 | P | 30 | -2 | ycf1;ycf1 | IRb;IRb |
|  | 34 | 114009 | 133499 | F | 30 | -2 | ycf1;ycf1 | IRb;IRa |
|  | 35 | 118045 | 118046 | P | 30 | -2 | IGS | SSC;SSC |
|  | 36 | 133499 | 133499 | P | 30 | -2 | ycf1;ycf1 | IRa;IRa |
|  | 37 | 38462 | 48177 | P | 30 | -3 | trnS-UGA;trnS-GGA | LSC;LSC |
|  | 38 | 47036 | 102717 | F | 30 | -3 | ycf3;IGS | LSC;IRb |
|  | 39 | 47036 | 144791 | P | 30 | -3 | ycf3;IGS | LSC;IRa |
|  | 40 | 74449 | 125476 | P | 30 | -3 | clpP;ndhA | LSC;SSC |
|  | 41 | 125480 | 125482 | P | 30 | -3 | ndhA;ndhA | SSC;SSC |
|  | 42 | 120223 | 120226 | R | 30 | -1 | IGS | SSC;SSC |
| *C. velutina* | 1 | 87353 | 87421 | F | 51 | -3 | rpl22;rpl22 | LSC;LSC |
|  | 2 | 92928 | 92949 | F | 47 | -3 | ycf2;ycf2 | IRb;IRb |
|  | 3 | 92928 | 154566 | P | 47 | -3 | ycf2;ycf2 | IRb;IRa |
|  | 4 | 92949 | 154587 | P | 47 | -3 | ycf2;ycf2 | IRb;IRa |
|  | 5 | 154566 | 154587 | F | 47 | -3 | ycf2;ycf2 | IRa;IRa |
|  | 6 | 95381 | 95399 | F | 46 | -1 | ycf2;ycf2 | IRb;IRb |
|  | 7 | 95381 | 152117 | P | 46 | -1 | ycf2;ycf2 | IRb;IRa |
|  | 8 | 95399 | 152135 | P | 46 | -1 | ycf2;ycf2 | IRb;IRa |
|  | 9 | 152117 | 152135 | F | 46 | -1 | ycf2;ycf2 | IRa;IRa |
|  | 10 | 102715 | 125140 | F | 42 | 0 | IGS;ndhA | IRb;SSC |
|  | 11 | 125140 | 144805 | P | 42 | 0 | ndhA;IGS | SSC;IRa |
|  | 12 | 47032 | 125139 | F | 42 | -3 | ycf3;ndhA | LSC;SSC |
|  | 13 | 42069 | 44293 | F | 41 | -3 | psaB;psaA | LSC;LSC |
|  | 14 | 47035 | 102717 | F | 39 | -2 | ycf3;IGS | LSC;IRb |
|  | 15 | 47035 | 144806 | P | 39 | -2 | ycf3;IGS | LSC;IRa |
|  | 16 | 87368 | 87436 | F | 36 | -2 | rpl22;rpl22 | LSC;LSC |
|  | 17 | 87339 | 87407 | F | 35 | -3 | IGS;rpl22 | LSC;LSC |
|  | 18 | 33912 | 33915 | P | 33 | -3 | IGS | LSC;LSC |
|  | 19 | 154581 | 154602 | F | 32 | -1 | ycf2;ycf2 | IRa;IRa |
|  | 20 | 9191 | 38471 | F | 32 | -3 | trnS-GCU;trnS-UGA | LSC;LSC |
|  | 21 | 108233 | 108271 | F | 31 | -2 | trnA-UGC;trnA-UGC | IRb;IRb |
|  | 22 | 108233 | 139260 | P | 31 | -2 | trnA-UGC;trnA-UGC | IRb;IRa |
|  | 23 | 108271 | 139298 | P | 31 | -2 | trnA-UGC;trnA-UGC | IRb;IRa |
|  | 24 | 121902 | 121905 | P | 31 | -2 | IGS | SSC;SSC |
|  | 25 | 139260 | 139298 | F | 31 | -2 | trnA-UGC;trnA-UGC | IRa;IRa |
|  | 26 | 95378 | 95414 | F | 31 | -3 | ycf2;ycf2 | IRb;IRb |
|  | 27 | 95378 | 152117 | P | 31 | -3 | ycf2;ycf2 | IRb;IRa |
|  | 28 | 95414 | 152153 | P | 31 | -3 | ycf2;ycf2 | IRb;IRa |
|  | 29 | 152114 | 152150 | F | 31 | -3 | ycf2;ycf2 | IRa;IRa |
|  | 30 | 7535 | 7537 | R | 31 | -3 | IGS | LSC;LSC |
|  | 31 | 9193 | 48188 | P | 30 | 0 | trnS-GCU;trnS-GGA | LSC;LSC |
|  | 32 | 49941 | 49945 | P | 30 | -2 | IGS | LSC;LSC |
|  | 33 | 114021 | 114021 | P | 30 | -2 | ycf1;ycf1 | IRb;IRb |
|  | 34 | 114021 | 133511 | F | 30 | -2 | ycf1;ycf1 | IRb;IRa |
|  | 35 | 118057 | 118058 | P | 30 | -2 | IGS | SSC;SSC |
|  | 36 | 133511 | 133511 | P | 30 | -2 | ycf1;ycf1 | IRa;IRa |
|  | 37 | 38473 | 48188 | P | 30 | -3 | trnS-UGA;trnS-GGA | LSC;LSC |
|  | 38 | 47047 | 102729 | F | 30 | -3 | ycf3;IGS | LSC;IRb |
|  | 39 | 47047 | 144803 | P | 30 | -3 | ycf3;IGS | LSC;IRa |
|  | 40 | 125492 | 125494 | P | 30 | -3 | ndhA;ndhA | SSC;SSC |
|  | 41 | 120235 | 120236 | R | 30 | -1 | IGS | SSC;SSC |
| *L. chinense* | 1 | 92951 | 92972 | F | 47 | -3 | ycf2;ycf2 | IRb;IRb |
|  | 2 | 92951 | 154587 | P | 47 | -3 | ycf2;ycf2 | IRb;IRa |
|  | 3 | 92972 | 154608 | P | 47 | -3 | ycf2;ycf2 | IRb;IRa |
|  | 4 | 154587 | 154608 | F | 47 | -3 | ycf2;ycf2 | IRa;IRa |
|  | 5 | 95404 | 95422 | F | 46 | -1 | ycf2;ycf2 | IRb;IRb |
|  | 6 | 95404 | 152138 | P | 46 | -1 | ycf2;ycf2 | IRb;IRa |
|  | 7 | 95422 | 152156 | P | 46 | -1 | ycf2;ycf2 | IRb;IRa |
|  | 8 | 152138 | 152156 | F | 46 | -1 | ycf2;ycf2 | IRa;IRa |
|  | 9 | 102735 | 125138 | F | 42 | -1 | IGS;ndhA | IRb;SSC |
|  | 10 | 125138 | 144829 | P | 42 | -1 | ndhA;IGS | SSC;IRa |
|  | 11 | 42073 | 44297 | F | 41 | -3 | psaB;psaA | LSC;LSC |
|  | 12 | 47039 | 102737 | F | 39 | -2 | ycf3;IGS | LSC;IRb |
|  | 13 | 47039 | 144830 | P | 39 | -2 | ycf3;IGS | LSC;IRa |
|  | 14 | 47039 | 125140 | F | 39 | -3 | ycf3;ndhA | LSC;SSC |
|  | 15 | 87356 | 87424 | F | 38 | -3 | IGS;rpl22 | LSC;LSC |
|  | 16 | 87389 | 87457 | F | 36 | -3 | rpl22;rpl22 | LSC;LSC |
|  | 17 | 39278 | 39287 | P | 35 | -3 | IGS | LSC;LSC |
|  | 18 | 154602 | 154623 | F | 32 | -1 | ycf2;ycf2 | IRa;IRa |
|  | 19 | 9244 | 38501 | F | 32 | -3 | trnS-GCU;trnS-UGA | LSC;LSC |
|  | 20 | 108245 | 108283 | F | 31 | -2 | trnA-UGC;trnA-UGC | IRb;IRb |
|  | 21 | 108245 | 139292 | P | 31 | -2 | trnA-UGC;trnA-UGC | IRb;IRa |
|  | 22 | 108283 | 139330 | P | 31 | -2 | trnA-UGC;trnA-UGC | IRb;IRa |
|  | 23 | 139292 | 139330 | F | 31 | -2 | trnA-UGC;trnA-UGC | IRa;IRa |
|  | 24 | 95401 | 95437 | F | 31 | -3 | ycf2;ycf2 | IRb;IRb |
|  | 25 | 95401 | 152138 | P | 31 | -3 | ycf2;ycf2 | IRb;IRa |
|  | 26 | 95437 | 152174 | P | 31 | -3 | ycf2;ycf2 | IRb;IRa |
|  | 27 | 152135 | 152171 | F | 31 | -3 | ycf2;ycf2 | IRa;IRa |
|  | 28 | 7600 | 7602 | R | 31 | -3 | IGS | LSC;LSC |
|  | 29 | 9246 | 48211 | P | 30 | 0 | trnS-GCU;trnS-GCU | LSC;LSC |
|  | 30 | 54148 | 54174 | F | 30 | -1 | IGS | LSC;LSC |
|  | 31 | 81717 | 81718 | P | 30 | -2 | IGS | LSC;LSC |
|  | 32 | 38503 | 48211 | P | 30 | -3 | trnS-UGA;trnS-GCU | LSC;LSC |
|  | 33 | 47051 | 102749 | F | 30 | -3 | ycf3;IGS | LSC;IRb |
|  | 34 | 47051 | 144827 | P | 30 | -3 | ycf3;IGS | LSC;IRa |
|  | 35 | 118042 | 118045 | P | 30 | -3 | IGS | SSC;SSC |
|  | 36 | 120221 | 120222 | R | 30 | -1 | IGS | SSC;SSC |

Table S4 Simple sequence repeats (SSRs) in the complete chloroplast genomes of *C*. *glandulifera*, *C*. *velutina*, and *L*. *chinense*.

| Species | Region | Number | Proportion | Exon | Intron | Intergenic | Type | Times | Number |
| --- | --- | --- | --- | --- | --- | --- | --- | --- | --- |
| *C. glandulifera* | All | 178 | 100.00% |  |  |  | A | 8 | 17 |
|  | IR | 31 | 17.40% | 22 | 4 | 5 | A | 9 | 20 |
|  | LSC | 127 | 71.30% | 30 | 19 | 78 | A | 10 | 11 |
|  | SSC | 20 | 11.20% | 10 | 1 | 9 | A | 11 | 4 |
|  |  |  |  |  |  |  | A | 12 | 5 |
|  |  |  |  |  |  |  | A | 13 | 2 |
|  |  |  |  |  |  |  | A | 14 | 4 |
|  |  |  |  |  |  |  | A | 15 | 1 |
|  |  |  |  |  |  |  | A | 18 | 1 |
|  |  |  |  |  |  |  | C | 8 | 1 |
|  |  |  |  |  |  |  | C | 11 | 1 |
|  |  |  |  |  |  |  | G | 8 | 2 |
|  |  |  |  |  |  |  | G | 9 | 1 |
|  |  |  |  |  |  |  | T | 8 | 19 |
|  |  |  |  |  |  |  | T | 9 | 22 |
|  |  |  |  |  |  |  | T | 10 | 24 |
|  |  |  |  |  |  |  | T | 11 | 7 |
|  |  |  |  |  |  |  | T | 12 | 3 |
|  |  |  |  |  |  |  | T | 13 | 1 |
|  |  |  |  |  |  |  | T | 14 | 4 |
|  |  |  |  |  |  |  | T | 15 | 1 |
|  |  |  |  |  |  |  | T | 19 | 1 |
|  |  |  |  |  |  |  | AT | 5 | 4 |
|  |  |  |  |  |  |  | TA | 5 | 2 |
|  |  |  |  |  |  |  | TA | 6 | 1 |
|  |  |  |  |  |  |  | TA | 7 | 1 |
|  |  |  |  |  |  |  | TC | 5 | 1 |
|  |  |  |  |  |  |  | AAC | 3 | 2 |
|  |  |  |  |  |  |  | AAG | 3 | 4 |
|  |  |  |  |  |  |  | AAT | 3 | 5 |
|  |  |  |  |  |  |  | ACC | 3 | 1 |
|  |  |  |  |  |  |  | AGA | 3 | 3 |
|  |  |  |  |  |  |  | AGC | 3 | 1 |
|  |  |  |  |  |  |  | AGT | 3 | 1 |
|  |  |  |  |  |  |  | ATA | 3 | 4 |
|  |  |  |  |  |  |  | ATA | 4 | 1 |
|  |  |  |  |  |  |  | ATC | 3 | 1 |
|  |  |  |  |  |  |  | ATG | 3 | 2 |
|  |  |  |  |  |  |  | ATT | 3 | 2 |
|  |  |  |  |  |  |  | CAA | 3 | 1 |
|  |  |  |  |  |  |  | CAG | 3 | 1 |
|  |  |  |  |  |  |  | CTC | 3 | 1 |
|  |  |  |  |  |  |  | CTG | 3 | 1 |
|  |  |  |  |  |  |  | CTT | 3 | 3 |
|  |  |  |  |  |  |  | GAA | 3 | 2 |
|  |  |  |  |  |  |  | GAA | 4 | 1 |
|  |  |  |  |  |  |  | GAT | 3 | 1 |
|  |  |  |  |  |  |  | GCA | 3 | 1 |
|  |  |  |  |  |  |  | GCT | 3 | 1 |
|  |  |  |  |  |  |  | GGA | 3 | 2 |
|  |  |  |  |  |  |  | GGT | 3 | 1 |
|  |  |  |  |  |  |  | GTT | 3 | 1 |
|  |  |  |  |  |  |  | TAA | 3 | 2 |
|  |  |  |  |  |  |  | TCA | 3 | 1 |
|  |  |  |  |  |  |  | TCT | 3 | 3 |
|  |  |  |  |  |  |  | TGA | 3 | 1 |
|  |  |  |  |  |  |  | TTA | 3 | 3 |
|  |  |  |  |  |  |  | TTA | 4 | 1 |
|  |  |  |  |  |  |  | TTC | 3 | 7 |
|  |  |  |  |  |  |  | TTG | 3 | 4 |
|  |  |  |  |  |  |  | AAAT | 3 | 1 |
|  |  |  |  |  |  |  | ATTT | 3 | 1 |
|  |  |  |  |  |  |  | GAAA | 3 | 1 |
|  |  |  |  |  |  |  | TGAA | 3 | 1 |
|  |  |  |  |  |  |  | TTCT | 3 | 1 |
|  |  |  |  |  |  |  | TATTT | 3 | 1 |
|  |  |  |  |  |  |  | TTCTA | 3 | 1 |
| *C. velutina* | All | 178 | 100.00% |  |  |  | A | 8 | 17 |
|  | IR | 31 | 17.40% | 21 | 4 | 6 | A | 9 | 22 |
|  | LSC | 127 | 71.30% | 30 | 19 | 78 | A | 10 | 11 |
|  | SSC | 20 | 11.20% | 10 | 1 | 9 | A | 11 | 3 |
|  |  |  |  |  |  |  | A | 12 | 4 |
|  |  |  |  |  |  |  | A | 13 | 3 |
|  |  |  |  |  |  |  | A | 14 | 3 |
|  |  |  |  |  |  |  | A | 15 | 1 |
|  |  |  |  |  |  |  | A | 18 | 1 |
|  |  |  |  |  |  |  | C | 8 | 1 |
|  |  |  |  |  |  |  | C | 11 | 1 |
|  |  |  |  |  |  |  | G | 8 | 2 |
|  |  |  |  |  |  |  | G | 10 | 1 |
|  |  |  |  |  |  |  | T | 8 | 19 |
|  |  |  |  |  |  |  | T | 9 | 22 |
|  |  |  |  |  |  |  | T | 10 | 21 |
|  |  |  |  |  |  |  | T | 11 | 10 |
|  |  |  |  |  |  |  | T | 12 | 3 |
|  |  |  |  |  |  |  | T | 13 | 1 |
|  |  |  |  |  |  |  | T | 14 | 5 |
|  |  |  |  |  |  |  | T | 19 | 1 |
|  |  |  |  |  |  |  | AT | 5 | 4 |
|  |  |  |  |  |  |  | TA | 5 | 3 |
|  |  |  |  |  |  |  | TA | 7 | 1 |
|  |  |  |  |  |  |  | TC | 5 | 1 |
|  |  |  |  |  |  |  | AAC | 3 | 2 |
|  |  |  |  |  |  |  | AAG | 3 | 4 |
|  |  |  |  |  |  |  | AAT | 3 | 5 |
|  |  |  |  |  |  |  | ACC | 3 | 1 |
|  |  |  |  |  |  |  | AGA | 3 | 3 |
|  |  |  |  |  |  |  | AGC | 3 | 1 |
|  |  |  |  |  |  |  | AGT | 3 | 1 |
|  |  |  |  |  |  |  | ATA | 3 | 4 |
|  |  |  |  |  |  |  | ATA | 4 | 1 |
|  |  |  |  |  |  |  | ATC | 3 | 1 |
|  |  |  |  |  |  |  | ATG | 3 | 2 |
|  |  |  |  |  |  |  | ATT | 3 | 2 |
|  |  |  |  |  |  |  | CAA | 3 | 1 |
|  |  |  |  |  |  |  | CAG | 3 | 1 |
|  |  |  |  |  |  |  | CTC | 3 | 1 |
|  |  |  |  |  |  |  | CTG | 3 | 1 |
|  |  |  |  |  |  |  | CTT | 3 | 3 |
|  |  |  |  |  |  |  | GAA | 3 | 2 |
|  |  |  |  |  |  |  | GAT | 3 | 1 |
|  |  |  |  |  |  |  | GCA | 3 | 1 |
|  |  |  |  |  |  |  | GCT | 3 | 1 |
|  |  |  |  |  |  |  | GGA | 3 | 2 |
|  |  |  |  |  |  |  | GGT | 3 | 1 |
|  |  |  |  |  |  |  | GTT | 3 | 1 |
|  |  |  |  |  |  |  | TAA | 3 | 2 |
|  |  |  |  |  |  |  | TCA | 3 | 1 |
|  |  |  |  |  |  |  | TCT | 3 | 3 |
|  |  |  |  |  |  |  | TGA | 3 | 1 |
|  |  |  |  |  |  |  | TTA | 3 | 3 |
|  |  |  |  |  |  |  | TTA | 4 | 1 |
|  |  |  |  |  |  |  | TTC | 3 | 7 |
|  |  |  |  |  |  |  | TTG | 3 | 4 |
|  |  |  |  |  |  |  | AAAT | 3 | 1 |
|  |  |  |  |  |  |  | ATTT | 3 | 1 |
|  |  |  |  |  |  |  | GAAA | 3 | 1 |
|  |  |  |  |  |  |  | TGAA | 3 | 1 |
|  |  |  |  |  |  |  | TTCT | 3 | 1 |
|  |  |  |  |  |  |  | TATTT | 3 | 1 |
|  |  |  |  |  |  |  | TTCTA | 3 | 1 |
| *L. chinense* | All | 175 | 100.00% |  |  |  | A | 8 | 20 |
|  | IR | 29 | 16.60% | 19 | 4 | 6 | A | 9 | 19 |
|  | LSC | 126 | 72.00% | 32 | 18 | 76 | A | 10 | 8 |
|  | SSC | 20 | 11.40% | 10 | 1 | 9 | A | 11 | 4 |
|  |  |  |  |  |  |  | A | 12 | 4 |
|  |  |  |  |  |  |  | A | 13 | 3 |
|  |  |  |  |  |  |  | A | 14 | 1 |
|  |  |  |  |  |  |  | A | 15 | 2 |
|  |  |  |  |  |  |  | A | 16 | 1 |
|  |  |  |  |  |  |  | C | 8 | 1 |
|  |  |  |  |  |  |  | C | 9 | 1 |
|  |  |  |  |  |  |  | G | 8 | 1 |
|  |  |  |  |  |  |  | G | 11 | 1 |
|  |  |  |  |  |  |  | T | 8 | 18 |
|  |  |  |  |  |  |  | T | 9 | 21 |
|  |  |  |  |  |  |  | T | 10 | 14 |
|  |  |  |  |  |  |  | T | 11 | 11 |
|  |  |  |  |  |  |  | T | 12 | 3 |
|  |  |  |  |  |  |  | T | 13 | 4 |
|  |  |  |  |  |  |  | T | 14 | 4 |
|  |  |  |  |  |  |  | T | 15 | 1 |
|  |  |  |  |  |  |  | T | 16 | 1 |
|  |  |  |  |  |  |  | AT | 5 | 3 |
|  |  |  |  |  |  |  | TA | 5 | 4 |
|  |  |  |  |  |  |  | TA | 6 | 1 |
|  |  |  |  |  |  |  | TA | 9 | 1 |
|  |  |  |  |  |  |  | TC | 5 | 1 |
|  |  |  |  |  |  |  | AAC | 3 | 2 |
|  |  |  |  |  |  |  | AAG | 3 | 3 |
|  |  |  |  |  |  |  | AAT | 3 | 2 |
|  |  |  |  |  |  |  | AAT | 4 | 1 |
|  |  |  |  |  |  |  | ACC | 3 | 1 |
|  |  |  |  |  |  |  | AGA | 3 | 4 |
|  |  |  |  |  |  |  | AGC | 3 | 1 |
|  |  |  |  |  |  |  | AGT | 3 | 1 |
|  |  |  |  |  |  |  | ATA | 3 | 2 |
|  |  |  |  |  |  |  | ATA | 4 | 2 |
|  |  |  |  |  |  |  | ATC | 3 | 1 |
|  |  |  |  |  |  |  | ATG | 3 | 2 |
|  |  |  |  |  |  |  | ATT | 3 | 1 |
|  |  |  |  |  |  |  | CAA | 3 | 1 |
|  |  |  |  |  |  |  | CAG | 3 | 2 |
|  |  |  |  |  |  |  | CTC | 3 | 1 |
|  |  |  |  |  |  |  | CTG | 3 | 1 |
|  |  |  |  |  |  |  | CTT | 3 | 3 |
|  |  |  |  |  |  |  | GAA | 3 | 2 |
|  |  |  |  |  |  |  | GAT | 3 | 1 |
|  |  |  |  |  |  |  | GCA | 3 | 1 |
|  |  |  |  |  |  |  | GCT | 3 | 1 |
|  |  |  |  |  |  |  | GGA | 3 | 1 |
|  |  |  |  |  |  |  | GGT | 3 | 1 |
|  |  |  |  |  |  |  | TAA | 3 | 1 |
|  |  |  |  |  |  |  | TAA | 4 | 1 |
|  |  |  |  |  |  |  | TCA | 3 | 1 |
|  |  |  |  |  |  |  | TCT | 3 | 3 |
|  |  |  |  |  |  |  | TGA | 3 | 1 |
|  |  |  |  |  |  |  | TTA | 3 | 1 |
|  |  |  |  |  |  |  | TTA | 4 | 1 |
|  |  |  |  |  |  |  | TTC | 3 | 7 |
|  |  |  |  |  |  |  | TTG | 3 | 4 |
|  |  |  |  |  |  |  | AAAT | 3 | 1 |
|  |  |  |  |  |  |  | ATAA | 4 | 1 |
|  |  |  |  |  |  |  | TAAA | 3 | 1 |
|  |  |  |  |  |  |  | TTTA | 3 | 1 |
|  |  |  |  |  |  |  | TATTT | 3 | 1 |
|  |  |  |  |  |  |  | TCTTT | 3 | 1 |
|  |  |  |  |  |  |  | CTAAGA | 3 | 1 |
